# Clinical frailty, and not features of acute infection, is associated with late mortality in COVID‐19: a retrospective cohort study

**DOI:** 10.1002/jcsm.12966

**Published:** 2022-03-07

**Authors:** Nikolaos I. Vlachogiannis, Kenneth F. Baker, Georgios Georgiopoulos, Charalampos Lazaridis, Ina Schim van der Loeff, Aidan T. Hanrath, Kateryna Sopova, Simon Tual‐Chalot, Aikaterini Gatsiou, Ioakim Spyridopoulos, Kimon Stamatelopoulos, Christopher J.A. Duncan, Konstantinos Stellos

**Affiliations:** ^1^ Biosciences Institute, Vascular Biology and Medicine Theme, Faculty of Medical Sciences Newcastle University Newcastle Upon Tyne UK; ^2^ RVI and Freeman Hospitals Newcastle upon Tyne Hospitals NHS Foundation Trust Newcastle Upon Tyne UK; ^3^ Translational and Clinical Research Institute Newcastle University Newcastle Upon Tyne UK; ^4^ NIHR Newcastle Biomedical Research Centre Newcastle University and Newcastle upon Tyne Hospitals NHS Foundation Trust Newcastle Upon Tyne UK; ^5^ Department of Clinical Therapeutics National and Kapodistrian University of Athens Medical School Athens Greece; ^6^ Department of Cardiovascular Research, European Center for Angioscience (ECAS) Heidelberg University Mannheim Germany; ^7^ German Centre for Cardiovascular Research (DZHK), partner site Heidelberg/Mannheim Mannheim Germany

**Keywords:** COVID‐19, Late mortality, Frailty, 4C mortality score, Prognosis

## Abstract

**Background:**

Coronavirus disease 2019 (COVID‐19) is associated with excess mortality after hospital discharge. Identification of patients at increased risk of death following hospital discharge is needed to guide clinical monitoring and early intervention. Herein, we aimed to identify predictors of early vs. late mortality in COVID‐19 patients.

**Methods:**

A total of 471 patients with polymerase chain reaction‐confirmed COVID‐19 were followed up for 9 months [median (inter‐quartile range) of follow‐up time: 271 (14) days] after hospital admission. COVID‐19‐related signs and symptoms, laboratory features, co‐morbidities, Coronavirus Clinical Characterisation Consortium (4C) mortality and Clinical Frailty Scale (CFS) scores were analysed by logistic regression for association with early (28 day) vs. late mortality. Receiver operating characteristic (ROC) analysis was used to determine the discriminative value of 4C and CFS scores for early vs. late mortality.

**Results:**

A total of 120 patients died within 28 days from hospital admission. Of the remaining 351 patients, 41 died within the next 8 months. Respiratory failure, systemic inflammation, and renal impairment were associated with early mortality, while active cancer and dementia were associated with late mortality, after adjustment for age and sex. 4C mortality score and CFS were associated with both early [odds ratio (OR) (95% confidence interval—CI): *4C*: 1.34 (1.25–1.45); *CFS*: 1.49 (1.33–1.66)] and late [OR (95% CI): *4C*: 1.23 (1.12–1.36); *CFS*: 2.04 (1.62–2.56)] mortality. After adjustment for CFS, the association between 4C and late mortality was lost. By ROC analysis, 4C mortality score was superior to CFS for 28 day mortality [area under the curve (AUC) (95% CI): 0.779 (0.732–0.825) vs. 0.723 (0.673–0.773), respectively; *P* = 0.039]. In contrast, CFS had higher predictive value for late mortality compared with 4C mortality score [AUC (95% CI): 0.830 (0.776–0.883) vs. 0.724 (0.650–0.798), respectively; *P* = 0.007].

**Conclusions:**

In our cohort, late mortality in COVID‐19 patients is more strongly associated with premorbid clinical frailty than with severity of the acute infection phase.

## Introduction

As of February 2022, the ongoing coronavirus disease 2019 (COVID‐19) pandemic has affected more than 400 million people and has caused more than 5.5 million deaths worldwide. Clinical severity of the disease can vary from mild, common cold symptoms to severe pneumonia, systemic inflammatory response syndrome, respiratory failure, and death. Thus, early and accurate patient risk stratification to guide clinical decision making is of utmost importance. Towards this goal, many clinical algorithms have been developed and validated in real‐life settings, most notably the Coronavirus Clinical Characterisation Consortium (4C) mortality score.^2^ However, such algorithms focus upon initial treatment escalation and short‐term prognosis, while longer‐term effects of COVID‐19 are now increasingly recognized.

Recent studies have shown that patients who recover from acute infection by the severe acute respiratory syndrome coronavirus 2 (SARS‐CoV‐2) virus exhibit continuous symptoms such as fatigue, muscle weakness, sleep and psychological disturbances,^3^ as well as increased incidence of new‐onset organ dysfunction, rates of hospitalizations, and mortality over subsequent months.^4^, ^5^ In a recent nationwide UK study, 30% of discharged COVID‐19 patients were readmitted to hospital over a 5 month period after discharge, while 1 out of 10 patients died.^4^ Whether this late morbidity and mortality is directly associated with COVID‐19 (‘post‐COVID syndrome’) or stems from deterioration of underlying co‐morbidities remains largely unknown. Therefore, identification of patients at risk of developing multi‐organ dysfunction or death after hospital discharge is needed to guide clinical monitoring and early intervention. Herein, we examined the clinical and laboratory predictors of early (28 day) vs. late mortality (>28 day till max. follow‐up period) among patients with COVID‐19 treated in a large UK tertiary academic medical centre. The main goals of our manuscript were to compare predictors of early (28 day) vs. late mortality in patients with COVID‐19; to examine whether 4C mortality score had a prognostic value beyond the first 28 days; and to compare the value of the well‐established 4C mortality score and Clinical Frailty Scale (CFS) for early (28 day) mortality vs. late mortality. We hypothesized that frailty is more important for late mortality (>28 day) compared with parameters related to the acute infectious disease.

## Methods

### Patient cohort

This is a retrospective analysis of real‐world clinical data captured during the first‐wave period of the COVID‐19 pandemic in the UK. In the initial few weeks of the pandemic (the so‐called ‘containment phase’), all patients testing positive for COVID‐19 were admitted to a high consequence infectious diseases (HCID) centre—which included our hospital—regardless of disease severity. A total of eight patients were admitted during the containment phase period, all of whom were young with mild disease and all of whom survived. Following this period, only patients with physiological perturbations requiring inpatient care were admitted to hospital. The decision to admit was made on an individual basis by the medical team rather than on a priori criteria, based on a severity of disease sufficient to warrant inpatient treatment (typically based upon the need for supplemental oxygen and/or intravenous therapy, and/or the requirement for intensive monitoring). Reflecting this high disease severity, 45% of the patients had an oxygen saturation <92% on admission, and 48% were tachypnoeic (respiratory rate >20). The observed high 28 day mortality rate (120/471, 25%) also underscores the high severity of infection in our cohort.

Electronic patient records were searched to identify all patients admitted with COVID‐19 to the Newcastle upon Tyne Hospitals NHS Foundation Trust between 31 January and 31 May 2020 inclusive. Only patients with a positive SARS‐CoV‐2 reverse transcription polymerase chain reaction (PCR) test based on combined nose and throat swab or sputum sample were included. We excluded patients under the age of 18 years, those who were already inpatients at the time of infection, or those who were asymptomatic and admitted for an unrelated reason (e.g. positive screening swab prior to surgery). Electronic clinical records were retrospectively reviewed to collect demographic, clinical, and laboratory data, with the aid of a version‐controlled data collection template (Excel, Microsoft Corporation). Mortality data for participants who were discharged alive were collected in January 2021, 271 (14) [median (inter‐quartile range—IQR)] days after admission. All deaths, including deaths in the community after hospital discharge, were captured by robust daily electronic system updates via primary care. The clinical management and 28 day outcomes of the first 316 patients admitted in this cohort have been previously published.^6^

### Risk score calculation

The CFS^7^, ^8^ is a widely used clinical algorithm with values ranging from 1 (very fit, among fittest persons for their age) to 9 (terminally ill, life expectancy <6 months) (*Table* S1). A cut‐off of 5 differentiates people who do not need help with everyday activities (CFS 0–4) from individuals who are dependent on others for everyday help to terminally ill patients (CFS 5–9)^8^ (*Table* S1). Therefore, a cut‐off of 5 was used in our analyses to define frailty. In accordance with the official guidance, we estimated CFS corresponding to the patients' clinical condition 2 weeks prior to acute illness (COVID‐19).^8^ More specifically, CFS at 2 weeks prior to admission was estimated by the admitting medical team based on available information in the medical records, supplemented by additional information from relatives or care home staff where applicable/available. The notes review was performed by qualified medical doctors (medical degree holders, registered with the General Medical Council, and working within the National Health Service). Where CFS was not documented in the medical notes, we retrospectively estimated CFS based on the information available in the medical records. All available information in the notes was used to estimate the CFS—this includes contemporaneous documentation in the medical and nursing admission notes as to physical function, assistance required for feeding/washing/dressing (all of which are mandatory fields on the nursing admission documentation at our hospital), previous clinic letters, social worker entries, and any documented discussions with relatives and/or carers. While CFS has been widely validated for individuals older than 65 years,^8^ a recent study showed that CFS can also predict in‐hospital death in younger individuals with COVID‐19.^9^ Therefore, we calculated CFS for all study participants.

Finally, we calculated the 4C mortality score^2^ for each patient at admission. 4C mortality score is a validated risk score to estimate risk of mortality in patients with COVID‐19. It consists of eight weighted variables (age, sex, co‐morbidities as defined in the Charlson Comorbidity Index^10^ with the addition of obesity, respiratory rate, oxygen saturation on room air, Glasgow Coma Scale score, urea, and C‐reactive protein at the point of hospital admission)^2^ (*Table* S2). Patients who were receiving supplementary oxygen at time of admission were considered as hypoxic (SpO_2_ < 92%). When data in one of the eight weighted variables were unavailable, 4C mortality score was not calculated. In total, 4C mortality score was calculated in 435/471 (92.4%) patients. Based on a cumulative score (0–21), patients are stratified in low (0–3), intermediate (4–8), high (9–14), and very high (≥15) risk for in‐hospital mortality.^2^

Inpatients were examined every day up to and including 28 days after admission, and an adapted World Health Organization ordinal scale for COVID‐19 was calculated. More specifically, this score has values from 1 to 8, with 8 corresponding to patients who are not hospitalized and do not require oxygen supplementation and 1 corresponding to death. For our analysis, we used a cut‐off of 4, which discriminates those patients who need hospitalization and oxygen supplementation.

### Statistical analysis

Normality of variable distribution was tested by Kolmogorov–Smirnov test. Continuous variables are presented as median (IQR) and categorical variables as absolute count (percentage). Pairwise comparisons between continuous variables were performed by Mann–Whitney *U* test, while Fisher's exact test was used to compare categorical variables between contrast groups. Logistic regression was used to examine the association of patients' signs and symptoms, as well as laboratory parameters, with early (28 day) and late (after 28 days) mortality, after controlling for age and sex. We further constructed receiver operating characteristic (ROC) curves for the occurrence of 28 day and late mortality across a range of values for continuous classifiers. To infer about superiority in discrimination between predictors of common outcome, we compared the corresponding area under the curve (AUC) using the trapezoidal rule. For comparison of independent ROC curves (i.e. prediction of the occurrence of 28 day and late mortality by the same continuous predictor), we followed the approach described by Gönen.^11^

To guard against overfitting, we used two distinct set of confounders, one composed of clinical variables and another integrating laboratory parameters. This approach reduced the overall number of covariates in the final multivariable model. Only variables with biological plausibility and/or statistical significance in univariable analysis were included in the final multivariable model. Thus, we ensured a ratio of 5–10 events per covariate used in the multivariable regression model. Moreover, we assessed whether addition of CFS significantly contributed to the core set of clinical or laboratory variables (core model) by calculating (i) explained variation (adjusted McFadden's *R* ^2^ index)^12^ (*Table* S3) and (ii) the Akaike information criterion^13^, ^14^, ^15^ and likelihood ratio tests for nested models with respect to 28 day and late mortality (*Table* S4). We also employed a cross‐validation procedure (iterated 50 times) by random splitting of the study population to training and validation data set and calculated the AUC of the final multivariable model in the two data sets^16^ (*Table* S5). Then, we compared AUCs between the two data sets and checked for concordance, which indicates absence of overfitting.

We used restricted cubic splines with three knots—fixed at the 10th, 50th, and 90th percentile of the underlying distribution of CFS or 4C mortality score—to model the association of these scores as continuous variables with overall mortality (dose–response curves). The Wald test was used to assess the significance of the non‐linear term. Finally, to increase the robustness of our results (internal validation), we employed resampling techniques and calculated normal‐based confidence intervals after bootstrapping with 1000 replicates for (i) difference in AUC for 4C and CFS with respect to late mortality and (ii) coefficients of 4C and CFS in multivariable [including age, sex, and estimated glomerular filtration rate (eGFR)] logistic regression models of early and late mortality. All tests were two‐tailed; results were considered significant when *P* < 0.05. Statistical analysis was performed using SPSS v. 26 and Stata v. 13.

### Ethics

The study was registered as a clinical service evaluation with the Newcastle upon Tyne Hospitals NHS Foundation Trust (Reference 10040), was exempt from ethical approval, and was exempt from a need for patient consent as a study of COVID‐19 under Regulation 3(4) of the Health Service Control of Patient Information Regulations 2002 (March 2020). Analysis of anonymized healthcare data was approved by the Caldicott Guardian (References 7523 and 7595).

## Results

### Prognostic value of COVID‐19‐related clinical signs and symptoms for 28 day and late mortality

A total of 471 consecutive adults admitted to hospital with PCR‐confirmed COVID‐19 were included in our analysis. Clinical and laboratory features of individuals who survived COVID‐19 (*n* = 351) vs. patients who died during the first 28 days from hospital admission (*n* = 120; early mortality group), as well as patients who survived up to 9 months after hospital admission with COVID‐19 (*n* = 310) vs. those patients who died after the first 28 days (*n* = 41; late mortality group), are summarized in *Tables* S6 and S7. The study cohort was aged 74 (25) years [median (IQR)], had a small male predominance (52.7%), and consisted mainly of British, Irish, or other White ethnicity (85.8%).

Our analysis showed that patients who survived beyond 28 days from admission more frequently presented with classical common cold symptoms such as pharyngitis and myalgia (*Table* S6). Regarding patient demographics, we did not find an association between male sex and mortality, as previously described in the literature^2^; however, a strong association of age with both early [OR (95% CI): 1.07 (1.05–1.09) per 1 year increase] and late [OR (95% CI): 1.07 (1.04–1.10) per 1 year increase] mortality was observed (*Table* 1).

**Table 1:** Clinical signs, symptoms, and co‐morbidities as predictors of early (28 day) vs. late mortality

	Early mortality		Late mortality	
	OR (95% CI)^a^	*P*‐value	OR (95% CI)^a^	*P*‐value
Demographics				
Age (years)	**1.07 (1.05–1.09)**	**<0.001**	**1.07 (1.04–1.10)**	**<0.001**
Age >65 years	**6.58 (3.57–12.15)**	**<0.001**	**5.47 (2.24–13.37)**	**<0.001**
Male sex	1.19 (0.78–1.80)	0.419	0.79 (0.41–1.52)	0.477
Symptoms and signs at presentation				
*T* > 38°C or *T* < 36°C	1.11 (0.69–1.80)	0.664	1.15 (0.55–2.42)	0.709
Cough	0.79 (0.51–1.23)	0.300	0.77 (0.39–1.52)	0.456
Sputum	0.65 (0.38–1.11)	0.113	0.85 (0.39–1.86)	0.679
Shortness of breath	**1.56 (1.003–2.42)**	**0.049**	0.73 (0.38–1.40)	0.343
Haemoptysis^b^	—	—	0.74 (0.09–5.92)	0.774
Rhinorrhoea	0.37 (0.08–1.66)	0.196	1.15 (0.25–5.30)	0.856
Pharyngitis^b^	—	—	0.41 (0.09–1.77)	0.231
Myalgia	**0.37 (0.19–0.73)**	**0.004**	0.36 (0.13–1.05)	0.062
Arthralgia^b^	—	—	0.93 (0.11–7.62)	0.945
Fatigue	1.01 (0.66–1.54)	0.972	0.93 (0.47–1.82)	0.826
Headache^b^	0.60 (0.27–1.32)	0.202	—	—
Confusion	**2.68 (1.71–4.20)**	**<0.001**	**2.68 (1.33–5.40)**	**0.006**
Anosmia	0.53 (0.18–1.59)	0.259	0.77 (0.17–3.44)	0.734
Abdominal pain	0.69 (0.35–1.39)	0.301	0.72 (0.24–2.12)	0.546
Diarrhoea	0.62 (0.34–1.11)	0.109	0.66 (0.27–1.63)	0.368
Heart rate (b.p.m.)	1.00 (0.99–1.01)	0.438	0.99 (0.98–1.01)	0.347
Heart rate >90 b.p.m.	1.13 (0.74–1.72)	0.573	0.52 (0.26–1.05)	0.069
Systolic BP (mmHg)	1.00 (0.99–1.01)	0.778	1.00 (0.99–1.02)	0.832
Diastolic BP (mmHg)	0.99 (0.98–1.01)	0.291	0.98 (0.95–1.00)	0.073
Respiratory rate (breaths per minute)	**1.07 (1.03–1.11)**	**<0.001**	1.00 (0.94–1.06)	0.966
RR > 20 (breaths per minute)	**2.07 (1.34–3.19)**	**0.001**	1.08 (0.56–2.11)	0.811
Hypoxia (SpO_2_ < 92%)	**2.22 (1.45–3.42)**	**<0.001**	1.19 (0.62–2.31)	0.598
Mean BP (mmHg)	1.00 (0.98–1.01)	0.674	0.99 (0.97–1.01)	0.386
Co‐morbidities				
Active cancer	1.60 (0.88–2.91)	0.122	**3.36 (1.49–7.58)**	**0.004**
Asthma	0.61 (0.30–1.26)	0.184	0.50 (0.15–1.69)	0.265
COPD	1.07 (0.61–1.90)	0.806	2.04 (0.93–4.47)	0.074
Other ILD	1.29 (0.52–3.20)	0.590	1.07 (0.24–4.90)	0.927
Ischaemic heart disease	1.28 (0.78–2.11)	0.332	**2.84 (1.41–5.73)**	**0.004**
Congestive heart failure	**2.93 (1.72–4.99)**	**<0.001**	1.90 (0.78–4.66)	0.160
Chronic kidney disease	**2.00 (1.26–3.16)**	**0.003**	1.71 (0.82–3.55)	0.149
DM diet‐controlled	1.47 (0.76–2.82)	0.253	0.79 (0.23–2.72)	0.708
DM tabs‐controlled	1.11 (0.58–2.14)	0.747	1.53 (0.60–3.93)	0.375
DM insulin	1.12 (0.48–2.61)	0.788	0.78 (0.17–3.47)	0.741
Hypertension	**2.14 (1.41–3.27)**	**<0.001**	1.19 (0.61–2.32)	0.619
Liver disease	0.29 (0.07–1.28)	0.103	1.44 (0.40–5.17)	0.576
Dementia	**2.60 (1.60–4.24)**	**<0.001**	**6.52 (3.19–13.33)**	**<0.001**
Adapted WHO ordinal scale at admission	**0.64 (0.49–0.82)**	**<0.001**	0.95 (0.64–1.41)	0.800
Adapted WHO ordinal scale at discharge	—	—	0.89 (0.58–1.38)	0.606

b.p.m., beats per minute; BP, blood pressure; CI, confidence interval; COPD, chronic obstructive pulmonary disease; DM, diabetes mellitus; ILD, interstitial lung disease; mmHg, millimetres of mercury; OR, odds ratio; RR, respiratory rate; *T*, temperature; WHO, World Health Organization. Values in bold denote statistically significant results.

^a^ Odds ratios are derived from binary logistic regression with early or late mortality as the dependent variable, and per 1‐unit increase for continuous variables, or vs. the reference category for categorical variables, as the independent variable.

^b^ OR not calculated because of zero occurrences in one of the groups.

**Figure 1:**
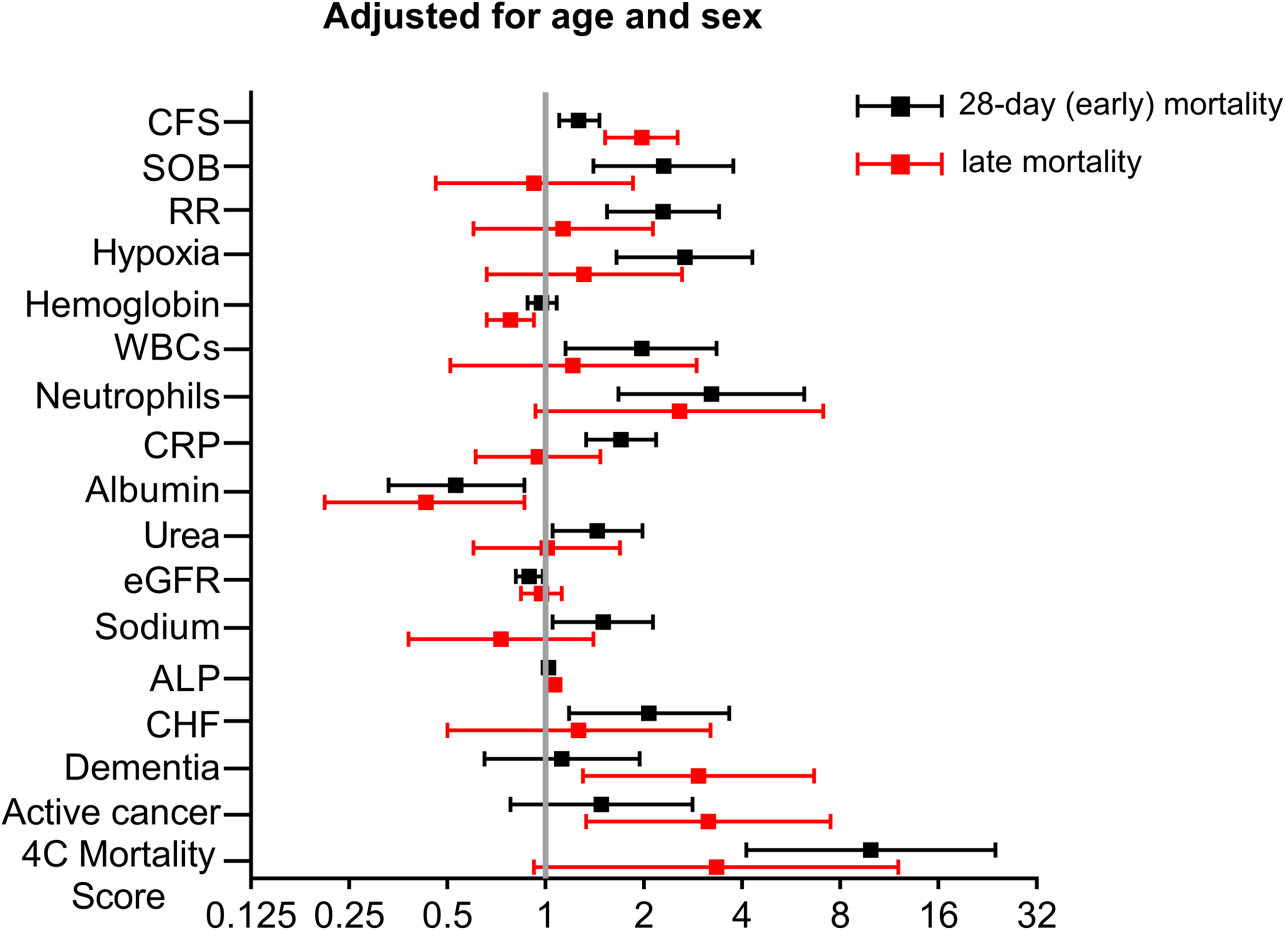
Forest plots showing odds ratios and 95% confidence intervals of independent predictors for early (28 day) and late mortality after adjustment for age and sex. Odds ratios correspond to 1‐unit increase in Clinical Frailty Scale (CFS) score, 10 breaths per minute increase in respiratory rate (RR), 10 g/L increase in haemoglobin levels, 10 × 10^9^/L increase in number of neutrophils, 100 mg/L increase in C‐reactive protein (CRP), 10 g/L increase in albumin, 10 mmol/L increase in urea levels, 10 mL/min/1.73 m^2^ increase in estimated glomerular filtration rate (eGFR), 10 mmol/L increase in sodium levels, and 10‐unit increase in 4C mortality score. OR for white blood cells (WBCs) corresponds to the presence of leukocytosis (>12 × 10^9^/L WBCs) or leukopenia (<4 × 10^9^/L WBCs). *X*‐axis is log_2_‐transformed for visual clarity. ALP, alkaline phosphatase; CHF, congestive heart failure; SOB, shortness of breath.

Respiratory distress, as evidenced by increased respiratory rate, shortness of breath, and decreased oxygen saturation at admission (SpO_2_ < 92%), was associated with increased early mortality (*Table* 1), a relationship that remained significant after adjustment for age and sex (*Table* 3 and *Figure* 1). In contrast, acute respiratory symptoms were not associated with late mortality. On the other hand, while confusion showed a significant association with both early and late mortality in univariate analyses (*Table* 1), no association was observed after adjustment for age and sex (*Table* 3).

In line with current evidence highlighting the presence of co‐morbidities as determining factor for COVID‐19 prognosis,^2^ we observed an association of arterial hypertension, congestive heart failure, and chronic kidney disease with early mortality (*Table* 1). On the other hand, active cancer and ischaemic heart were associated with late mortality (*Table* 1). Dementia was the only co‐morbid condition associated with both early and late mortality in univariate analyses (*Table* 1); however, after adjusting for age and sex, its association remained significant only with late mortality (*Table* 3 and *Figure* 1). Similarly, only heart failure and active cancer retained their association with early and late mortality, respectively, after controlling for the effect of age and sex (*Table* 3 and *Figure* 1). Further adjustment for renal function (eGFR), as a significant factor associated with mortality in patients with COVID‐19, did not significantly affect the results (*Table* S8).

### COVID‐19‐related laboratory parameters in the prediction of 28 day and late mortality

In addition to the clinical signs and symptoms, a number of laboratory features at point of admission were associated with increased mortality in our cohort. Presence of leucocytosis or leukopenia, elevated neutrophil count, elevated C‐reactive protein (CRP), decreased eGFR, increased urea, increased alkaline phosphatase (ALP), decreased albumin, and increased sodium were associated with 28 day mortality (*Table* 2). Of note, most of these associations remained unaffected by adjustment for age and sex: presence of leucocytosis/leukopenia, increased neutrophil count, elevated CRP, decreased eGFR, elevated urea, decreased albumin, and elevated sodium retained their association with 28 day mortality (*Table* 3 and *Figure* 1). In contrast, only ALP and the cachexia‐related markers haemoglobin and albumin were associated with late mortality after adjustment for age and sex (*Table* 3 and *Figure* 1). Further adjustment for renal function (eGFR), as a significant factor associated with mortality in patients with COVID‐19, did not significantly affect the results (*Table* S8).

**Table 2:** Laboratory features and risk scores as predictors of early (28 day) vs. late mortality

	Early mortality		Late mortality	
	OR (95% CI)^a^	*P*‐value	OR (95% CI)^a^	*P*‐value
Laboratory parameters				
Haemoglobin (g/L)	0.994 (0.985–1.003)	0.210	**0.972 (0.956–0.987)**	**<0.001**
WBCs (× 10^9^/L)	1.00 (0.98–1.02)	0.856	1.00 (0.98–1.03)	0.831
WBCs < 4 or WBCs > 12 (× 10^9^/L)	**2.06 (1.27–3.36)**	**0.003**	1.22 (0.53–2.79)	0.643
Neutrophils (× 10^9^/L)	**1.15 (1.09–1.23)**	**<0.001**	**1.14 (1.03–1.25)**	**0.008**
Eosinophils (× 10^9^/L)	0.01 (0.00–1.10)	0.055	13.3 (0.66–267)	0.091
Lymphocytes (× 10^9^/L)	0.97 (0.87–1.09)	0.622	0.99 (0.86–1.14)	0.853
Platelets (× 10^9^/L)	1.00 (0.999–1.003)	0.649	1.002 (0.999–1.006)	0.166
Urea (mmol/L)	**1.07 (1.04–1.11)**	**<0.001**	1.03 (0.98–1.07)	0.228
Creatinine (μmol/L)	1.001 (1.00–1.003)	0.166	1.00 (0.998–1.003)	0.718
eGFR (mL/min/1.73 m^2^)	**0.977 (0.968–0.985)**	**<0.001**	**0.986 (0.974–0.998)**	**0.027**
ALP (IU/L)	**1.003 (1.001–1.006)**	**0.011**	**1.007 (1.002–1.011)**	**0.003**
Bilirubin (μmol/L)	1.003 (0.988–1.018)	0.689	1.023 (0.996–1.051)	0.095
CRP (mg/L)	**1.004 (1.002–1.006)**	**<0.001**	0.999 (0.995–1.003)	0.595
Albumin (g/L)	**0.92 (0.88–0.96)**	**<0.001**	**0.90 (0.84–0.96)**	**0.001**
Sodium (mmol/L)	**1.07 (1.03–1.10)**	**<0.001**	0.99 (0.93–1.05)	0.729
Potassium (mmol/L)	1.36 (0.96–1.93)	0.083	0.96 (0.54–1.71)	0.900
Risk scores				
Clinical Frailty Scale	**1.49 (1.33–1.66)**	**<0.001**	**2.04 (1.62–2.56)**	**<0.001**
4C mortality score	**1.34 (1.25–1.45)**	**<0.001**	**1.23 (1.12–1.36)**	**<0.001**

ALP, alkaline phosphatase; CI, confidence interval; CRP, C‐reactive protein; eGFR, estimated glomerular filtration rate; OR, odds ratio; WBCs, white blood cells. Values in bold denote statistically significant results.

^a^ Odds ratios are derived from binary logistic regression with early or late mortality as the dependent variable, and per 1‐unit change in the variable depicted in each row as the independent variable.

**Table 3:** Association of disease features with early (28 day) and late mortality adjusted for age and sex

	Early mortality		Late mortality	
	OR (95% CI)^a^	*P*‐value	OR (95% CI)^a^	*P*‐value
Symptoms and signs at presentation				
Shortness of breath	**2.30 (1.40–3.76)**	**0.001**	0.92 (0.46–1.85)	0.819
Myalgia	0.74 (0.35–1.55)	0.425	0.80 (0.25–2.52)	0.702
Confusion	1.30 (0.78–2.14)	0.314	1.30 (0.60–2.81)	0.506
Respiratory rate (breaths per minute)	**1.09 (1.04–1.13)**	**<0.001**	1.01 (0.95–1.08)	0.716
RR > 20 (breaths per minute)	**2.53 (1.57–4.09)**	**<0.001**	1.23 (0.61–2.46)	0.562
Hypoxia (SpO_2_ < 92%)	**2.67 (1.65–4.30)**	**<0.001**	1.31 (0.66–2.62)	0.444
Co‐morbidities				
Active cancer	1.48 (0.78–2.82)	0.231	**3.15 (1.33–7.47)**	**0.009**
Ischaemic heart disease	0.82 (0.48–1.40)	0.460	1.84 (0.89–3.83)	0.102
Congestive heart failure	**2.07 (1.18–3.65)**	**0.011**	1.26 (0.50–3.20)	0.624
Chronic kidney disease	1.05 (0.64–1.74)	0.844	1.01 (0.47–2.18)	0.976
Hypertension	1.40 (0.89–2.21)	0.149	0.73 (0.36–1.49)	0.393
Dementia	1.12 (0.65–1.94)	0.671	**2.94 (1.30–6.65)**	**0.010**
Adapted WHO ordinal scale at admission	**0.49 (0.36–0.67)**	**<0.001**	0.90 (0.55–1.46)	0.661
Laboratory parameters				
Haemoglobin (g/L)	0.997 (0.987–1.008)	0.608	**0.975 (0.959–0.991)**	**0.003**
WBCs < 4 or WBCs > 12 (× 10^9^/L)	**1.97 (1.15–3.34)**	**0.013**	1.21 (0.51–2.90)	0.661
Neutrophils (× 10^9^/L)	**1.12 (1.05–1.20)**	**<0.001**	1.10 (0.99–1.22)	0.068
Urea (mmol/L)	**1.04 (1.00–1.07)**	**0.026**	1.00 (0.95–1.05)	0.972
eGFR (mL/min/1.73 m^2^)	**0.989 (0.979–0.998)**	**0.020**	1.00 (0.98–1.01)	0.674
ALP (IU/L)	1.002 (1.000–1.005)	0.075	**1.006 (1.002–1.011)**	**0.005**
CRP (mg/L)	**1.005 (1.003–1.008)**	**<0.001**	0.999 (0.996–1.004)	0.807
Albumin (g/L)	**0.94 (0.90–0.99)**	**0.011**	**0.92 (0.86–0.98)**	**0.017**
Sodium (mmol/L)	**1.04 (1.01–1.08)**	**0.025**	0.97 (0.91–1.03)	0.343
Risk scores				
Clinical Frailty Scale	**1.26 (1.10–1.46)**	**0.001**	**1.97 (1.52–2.54)**	**<0.001**
4C mortality score	**1.26 (1.15–1.37)**	**<0.001**	1.13 (0.99–1.28)	0.066

ALP, alkaline phosphatase; CI, confidence interval; CRP, C‐reactive protein; eGFR, estimated glomerular filtration rate; OR, odds ratio; RR, respiratory rate; WBCs, white blood cells; WHO, World Health Organization. Values in bold denote statistically significant results.

^a^ Odds ratios are derived from multivariable logistic regression with early or late mortality as the dependent variable, and per 1‐unit increase for continuous variables, or vs. the reference category for categorical variables, plus age and sex as independent variables.

### 4C mortality and Clinical Frailty Scale scores in risk stratification of hospitalized patients with COVID‐19 for early and late mortality

To examine the potential value of currently used COVID‐19 prognostic scores for late mortality, we calculated the 4C mortality score, an established and validated risk stratification tool for early (28 day) COVID‐19 mortality,^2^ and CFS,^7^ which has shown additive prognostic value over age and co‐morbidities in COVID‐19.^17^

Both 4C mortality score and CFS, which positively correlated with each other in our cohort (rho = 0.610, *P* < 0.001), were associated with total 9 month mortality [OR (95% CI) 4C: 1.34 (1.26–1.44), *P* < 0.001, and CFS: 1.72 (1.54–1.93), *P* < 0.001] (*Table* S9). By dose–response curves, we found a linear association for both CFS (*P* = 0.303 for the non‐linear term, OR for 80th to 20th percentile = 15.39, 95% CI 8.52–27.78, *P* < 0.001) and 4C (*P* = 0.215 for the non‐linear term, OR for 80th to 20th percentile = 9.17, 95% CI 5.17–16.26, *P* < 0.001) with overall mortality in patients with COVID‐19 (*Figure* S1).

**Figure 2:**
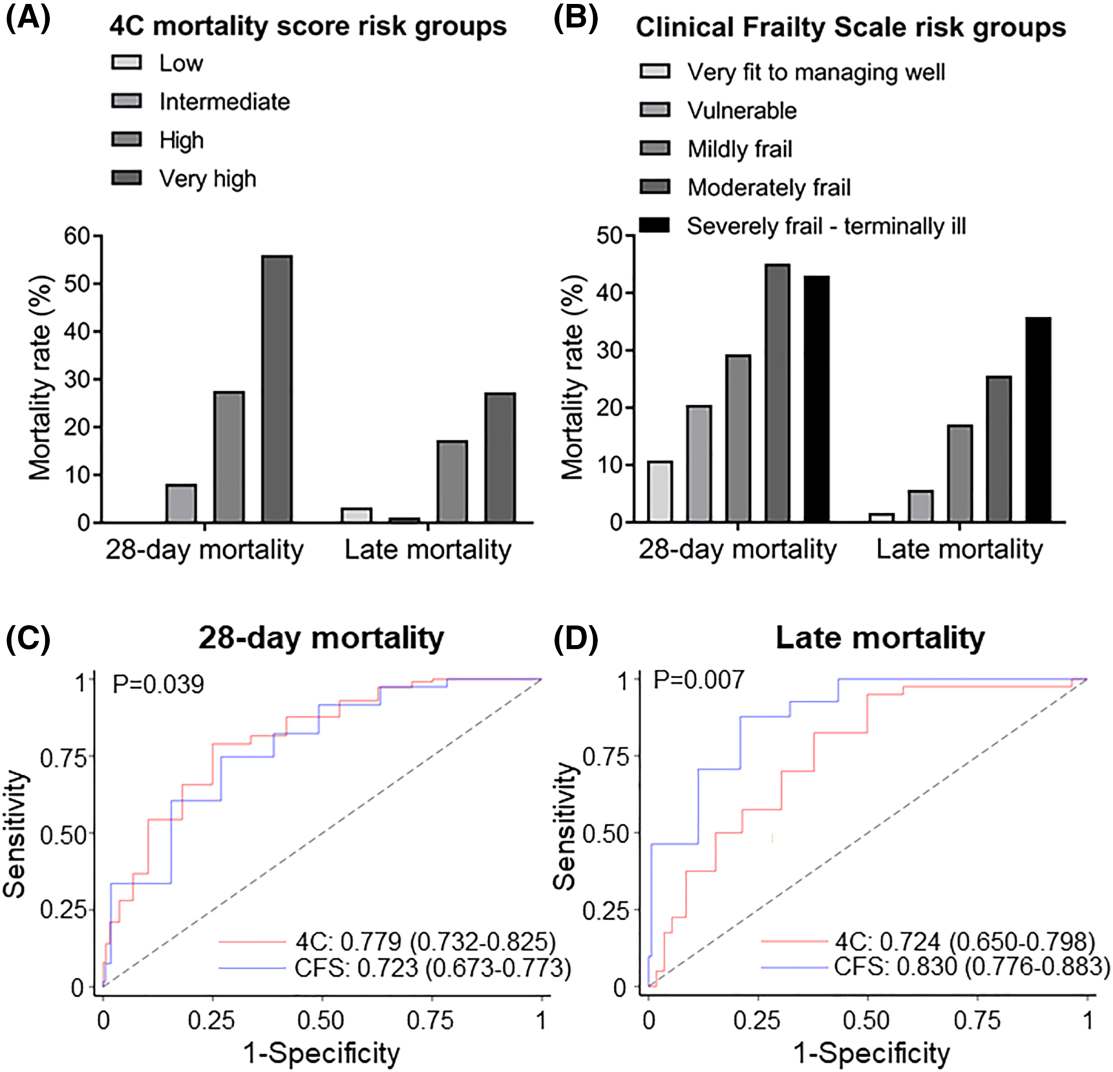
(A, B) Mortality rate in COVID‐19 patients according to 4C mortality score and Clinical Frailty Scale (CFS) risk groups. (A) Patients were classified according to 4C mortality score in four groups: low risk (4C: 0–3; *n* = 31), intermediate risk (4C: 4–8; *n* = 97), high risk (4C: 9–14; *n* = 232), and very high risk (4C: ≥15; *n* = 75). Bars represent mortality rate at 28 days after admission (early mortality) and between 29 days and 8 months (late mortality). (B) Early and late mortality according to CFS in five groups: very fit to managing well (CFS 1–3; *n* = 195), vulnerable (CFS 4; *n* = 44), mildly frail (CFS 5; *n* = 58), moderately frail (CFS 6; *n* = 71), and severely frail/terminally ill (CFS 7–9; *n* = 93). Receiver operating characteristic (ROC) curves and corresponding area(s) under the curve (AUC) for (C) early (28 day) and (D) late mortality in patients hospitalized with COVID‐19. The discriminatory performance of the 4C mortality and CFS scores for the occurrence of death is graphically assessed by corresponding ROC curves, and AUCs are compared with derive *P*‐values using the *χ* ^2^ distribution with one degree of freedom.

Of interest, we observed that a high 4C mortality score was associated with both early mortality [OR (95% CI): 1.34 (1.25–1.45)] and late mortality [OR (95% CI): 1.23 (1.12–1.36)] (*Table* 2 and *Figure* 2A). After adjusting for age and sex, 4C remained significantly associated with early mortality [adj. OR (95% CI): 1.26 (1.15–1.37), *P* < 0.001], but lost its association with late mortality [adj. OR (95% CI): 1.13 (0.99–1.28), *P* = 0.066] (*Table* 3). Similar results were observed after additional adjustment for eGFR (*Table* S8). After bootstrapping, similar results were observed, as 4C remained significantly associated with early mortality [OR (95% CI): 1.25 (1.14–1.37), *P* < 0.001], but lost its association with late mortality [OR (95% CI): 1.14 (0.996–1.31), *P* = 0.058].

Clinical Frailty Scale was also associated with both early and late mortality [OR (95% CI): 1.49 (1.33–1.66) and 2.04 (1.62–2.56) for early and late mortality, respectively] (*Table* 2 and *Figure* 2B). However, CFS retained its association with both early [adj. OR (95% CI): 1.26 (1.10–1.46), *P* = 0.001] and late mortality [adj. OR (95% CI): 1.97 (1.52–2.54), *P* < 0.001] after adjustment for age and sex (*Table* 3), or additional adjustment for eGFR (*Table* S8). After bootstrapping, similar results were observed [OR (95% CI) early mortality: 1.25 (1.06–1.47), *P* = 0.009, and late mortality: 1.96 (1.52–2.51), *P* < 0.001]. Finally, multivariable logistic regression analysis including all factors associated with early mortality (*Table* 4), late mortality (*Table* 5), or total 9 month mortality (*Table* S10) showed an independent association of CFS with all study outcomes. Importantly, after bootstrapping, the association of CFS with both 28 day and late mortality remained significant after controlling for clinical or laboratory variables [*28 day mortality*: OR (95% CI) for CFS after adjustment for clinical characteristics = 1.34 (1.12–1.59) and OR (95% CI) for CFS after adjustment for laboratory parameters = 1.33 (1.14–1.56)/*late mortality*: OR (95% CI) for CFS after adjustment for clinical characteristics = 1.99 (1.52–2.59) and OR (95% CI) for CFS after adjustment for laboratory parameters = 1.93 (1.49–2.49)]. Similar results were obtained when we used a cut‐off of 5 for CFS, which differentiates people who do not need help with everyday activities (CFS 0–4) from individuals who are dependent on others for everyday help to terminally ill patients (CFS 5–9) (*Tables* S11 and S12). Across the main multivariable logistic regression models for both 28 day and late mortality, no evidence of overfitting was shown (*Tables* S3–S5).

**Table 4:** Multivariable analysis of predictors for early (28 day) mortality

	OR (95% CI)^a^	*P*‐value
(a) Clinical characteristics		
Clinical Frailty Scale	**1.32 (1.13–1.55)**	**0.001**
Age >65 years	**3.31 (1.48–7.42)**	**0.004**
Shortness of breath	1.46 (0.81–2.61)	0.206
Myalgia	1.06 (0.46–2.45)	0.883
Confusion	1.58 (0.88–2.86)	0.127
RR > 20 (breaths per minute)	**2.01 (1.19–3.40)**	**0.009**
Hypoxia (SpO_2_ < 92%)	1.65 (0.95–2.85)	0.074
Congestive heart failure	**2.13 (1.17–3.90)**	**0.014**
Chronic kidney disease	1.21 (0.71–2.06)	0.481
Hypertension	**1.76 (1.06–2.91)**	**0.028**
Dementia	1.02 (0.54–1.93)	0.948
(b) Laboratory parameters		
Clinical Frailty Scale	**1.33 (1.16–1.53)**	**<0.001**
WBCs < 4 or WBCs > 12 (× 10^9^/L)	1.46 (0.77–2.79)	0.246
Neutrophils (× 10^9^/L)	1.07 (0.99–1.16)	0.103
Urea (mmol/L)	1.00 (0.96–1.05)	0.843
eGFR (mL/min/1.73 m^2^)	**0.986 (0.973–0.999)**	**0.031**
ALP (IU/L)	1.002 (0.999–1.005)	0.168
CRP (mg/L)	**1.004 (1.001–1.007)**	**0.007**
Albumin (g/L)	0.98 (0.93–1.04)	0.484
Sodium (mmol/L)	1.041 (0.998–1.085)	0.060

ALP, alkaline phosphatase; CI, confidence interval; CRP, C‐reactive protein; eGFR, estimated glomerular filtration rate; OR, odds ratio; RR, respiratory rate; WBCs, white blood cells. Values in bold denote statistically significant results.

^a^ Odds ratios are derived from multivariable logistic regression with early mortality as the dependent variable, and CFS as well as (a) all clinical features, or (b) all laboratory parameters associated at univariable analysis with early mortality, as independent variables.

**Table 5:** Multivariable analysis of predictors for late mortality

	OR (95% CI)^a^	*P*‐value
(a) Clinical characteristics		
Clinical Frailty Scale	**1.99 (1.50–2.63)**	**<0.001**
Age >65 years	0.79 (0.25–2.51)	0.691
Confusion	0.54 (0.20–1.46)	0.228
Active cancer	**4.35 (1.57–12.04)**	**0.005**
Ischaemic heart disease	1.95 (0.86–4.43)	0.112
Dementia	**2.85 (1.01–8.04)**	**0.048**
(b) Laboratory parameters		
Clinical Frailty Scale	**1.93 (1.48–2.51)**	**<0.001**
Haemoglobin (g/L)	0.981 (0.961–1.001)	0.056
Neutrophils (× 10^9^/L)	1.05 (0.94–1.18)	0.383
eGFR (mL/min/1.73 m^2^)	0.997 (0.981–1.013)	0.694
ALP (IU/L)	1.003 (0.998–1.009)	0.203
Albumin (g/L)	0.97 (0.89–1.05)	0.478

ALP, alkaline phosphatase; CI, confidence interval; eGFR, estimated glomerular filtration rate; OR, odds ratio. Values in bold denote statistically significant results.

^a^ Odds ratios are derived from multivariable logistic regression with late mortality as the dependent variable, and CFS as well as (a) all clinical features, or (b) all laboratory parameters associated at univariable analysis with late mortality, as independent variables.

**Figure 3:**
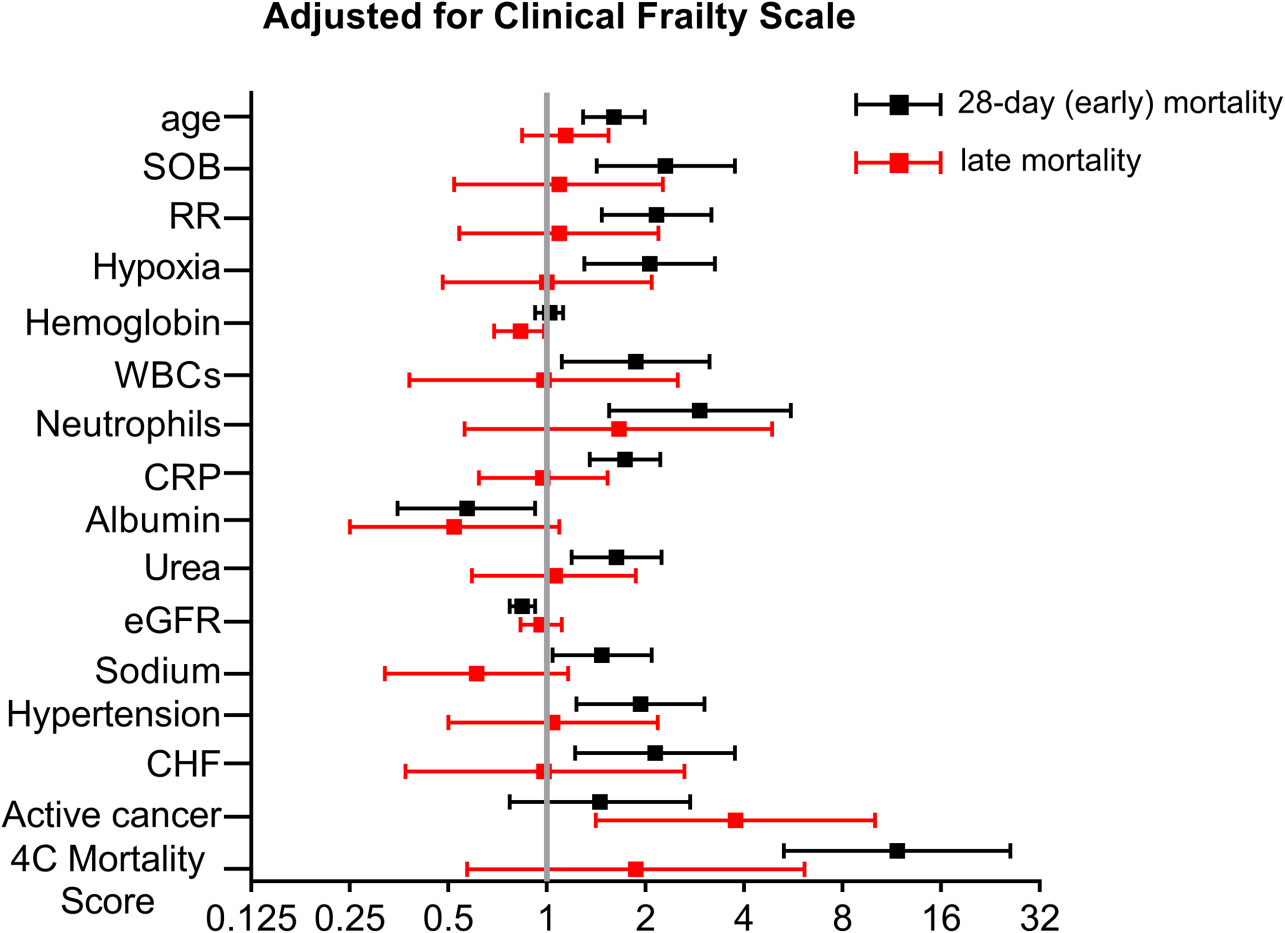
Forest plots showing odds ratios and 95% confidence intervals of independent predictors for early (28 day) and late mortality after adjustment for Clinical Frailty Scale. Odds ratios correspond to 10 year increase in age, 10 breaths per minute increase in respiratory rate (RR), 10 g/L increase in haemoglobin levels, 10 × 10^9^/L increase in number of neutrophils, 100 mg/L increase in reactive protein (CRP), 10 g/L increase in albumin, 10 mmol/L increase in urea levels, 10 mL/min/1.73 m^2^ increase in estimated glomerular filtration rate (eGFR), 10 mmol/L increase in sodium levels, and 10‐unit increase in 4C mortality score. OR for white blood cells (WBCs) corresponds to the presence of leukocytosis (>12 × 10^9^/L WBCs) or leukopenia (<4 × 10^9^/L WBCs). *X*‐axis is log_2_‐transformed for visual clarity. CHF, congestive heart failure; SOB, shortness of breath.

Next, by ROC analysis, we found that 4C mortality score and CFS had similar prognostic value for total 9 month mortality [4C AUC (95% CI): 0.781 (0.738–0.824) vs. CFS AUC (95% CI): 0.783 (0.740–0.826), *P* = 0.946] (*Figure* S2). On the other hand, 4C mortality score was superior to the CFS for predicting early mortality [4C AUC (95% CI): 0.779 (0.732–0.825) vs. CFS AUC (95% CI): 0.723 (0.673–0.773), *P* = 0.039; *Figure* 2C]. A reversed pattern was observed for late mortality, with the CFS outperforming the 4C mortality score [CFS AUC (95% CI): 0.830 (0.776–0.883) vs. 4C AUC (95% CI): 0.724 (0.650–0.798), *P* = 0.007, *Figure* 2D]. Moreover, CFS outperformed 4C for predicting late mortality after bootstrapping [OR (95% CI): 0.839 (0.820–0.846) vs. 0.726 (0.701–0.752), for CFS and 4C respectively, *P* < 0.001]. Accordingly, adjustment for CFS did not affect the relationship between age, respiratory symptoms, inflammation, and renal impairment at admission with 28 day mortality (*Table* S13 and *Figure* 3). On the other hand, only the presence of active cancer and low haemoglobin were independently associated with late mortality after adjustment for CFS (*Table* S13 and *Figure* 3), indicating the importance of frailty and active cancer as independent predictors of late mortality in this cohort.

Finally, a sensitivity analysis showed that CFS was significantly associated with both early and late mortality in participants aged >65 years, as well as in younger individuals (*Table* S14).

## Discussion

The main findings of the present study are as follows: (i) we show that late mortality, that is, in patients who survived the acute COVID‐19 infection, is not dependent on any of the characteristics of the disease course (inflammatory markers, clinical signs, and symptoms), but is only dependent on the premorbid status of the patient as quantified by CFS, and (ii) although 4C mortality score was indeed the best prognostic score for 28 day mortality, however, it should not probably be used for risk stratification of COVID‐19 survivors, as its prognostic value for late mortality was limited and significantly inferior to CFS.

Increasing evidence supports increased long‐term morbidity and mortality in COVID‐19 beyond the acute infection phase,^4^, ^5^ though the epidemiology of post‐COVID syndrome remains poorly defined. A recent study examining 47 780 patients who had survived acute COVID‐19 showed that discharged patients had 8 times higher risk of death (increasing to 14 times higher risk for individuals aged <70 years) compared with a general population control group matched for age, sex, ethnicity, and major co‐morbidities.^4^ Major complications among these patients included new‐onset organ dysfunction such as diabetes, kidney and liver disease, and especially major adverse cardiovascular events.^4^ While data on secondary prevention post‐COVID‐19 are conflicting, post‐discharge thromboprophylaxis significantly reduced thrombotic complications and all‐cause mortality, which were observed in approximately 7% of discharged COVID‐19 patients within 3 months.^5^ Thus, there remains an unmet need to identify patients who are at increased risk of developing severe organ dysfunction or death following hospital discharge, to facilitate planning of preventive measures and targeted rehabilitation. Despite matching for multiple risk factors, previous studies have not compared frailty status of patients, which CFS we show to be superior to 4C mortality score (including age, sex, co‐morbidities, and COVID‐19‐related symptoms) for late mortality prognosis, and therefore could partly account for the excess mortality observed in COVID‐19 patients.

Our study verifies the significance of COVID‐19‐related risk factors for in‐hospital mortality and validates the strong prognostic value of age and the 4C mortality score in the acute stage of disease. Respiratory dysfunction, strong acute inflammatory response, and renal impairment, along with major co‐morbidities, were the strongest age‐independent and sex‐independent predictors of early death in line with current evidence in literature.^2^, ^18^, ^19^, ^20^ On the other hand, we show that late mortality is not associated with the course of acute COVID‐19, which is further consolidated by the good clinical status of patients at the time of discharge. Baseline CFS score was the most significant prognostic factor for late mortality, supporting that late mortality is more strongly associated with premorbid performance status rather than specific COVID‐19‐related characteristics or infection course. Of interest, markers of cachexia such as low albumin and haemoglobin were also associated with late mortality independent of age and sex. Interestingly, frailty, malnutrition, and cachexia often co‐exist in older individuals; in a recent study examining more than 110 000 older individuals across the UK, frailty was detected in >90% of patients characterized by malnutrition and in 100% of patients characterized by cachexia,^21^ while recent studies suggest an inverse relationship between CFS and albumin or BMI.^22^ The association of CFS with various tissue loss syndromes (sarcopenia, frailty, cachexia, and malnutrition) as a general marker of baseline patient status is worth exploring in future studies regarding COVID‐19 patients and older individuals in general.

A meta‐analysis including 23 944 COVID‐19 patients showed that frailty is a major risk factor for COVID‐19 in‐hospital mortality.^23^ Frailty, measured with CFS, was associated with two‐fold to three‐fold increased risk of in‐hospital death by COVID‐19 independent of age, sex, and major co‐morbidities.^24^, ^25^ Apart from in‐hospital mortality, frailty has been associated with increased care needs after COVID‐19 discharge,^26^, ^27^ while in a recent study, CFS had additive prognostic value over age and SOFA score^28^ for cumulative 6 month mortality in 1830 patients with COVID‐19.^17^ Herein, we expand this observation by showing that CFS is superior to the widely used 4C mortality score for the prediction of late (after day 28) mortality. Of note, the association of CFS with late mortality was independent of age, underlining the importance of assessing the age‐related accumulation of clinical deficits (biological age) rather than chronological age, to develop personalized rehabilitation and monitoring plans.^29^ A nationwide British study showed that frailty was associated with increased risk of sepsis development following an infection consultation. Of note, severely frail patients aged 55 years had a similar probability of developing sepsis as non‐frail 85‐year‐olds, further underlining the significance of biological vs. chronological age.^30^ Frail patients (CFS ≥ 5) admitted in ICU with non‐COVID suspected infection had two‐fold increased risk of in‐hospital death, as well as two‐fold increased risk of long‐term care needs or rehospitalization within 30 days.^31^ Similarly, a 1.6‐fold to 3.5‐fold increase in late mortality (31 days to 2 years post‐infection) has also been documented in non‐COVID‐19 sepsis compared with uncomplicated infections or control population, respectively, matched for demographics, quality of life, and co‐morbidities.^32^

Future studies controlling for frailty scale are warranted to further support its independent prognostic role for late mortality in COVID‐19 and other severe infectious diseases. Finally, it is worth noting that frailty has been recognized as a modifier of all acute diseases. Frail individuals admitted in ICU due to various illnesses were more likely to die in hospital, or within the next 6–12 months.^33^, ^34^ Moreover, pre‐hospital frailty was associated with increased post‐hospital disability,^35^ with frail survivors being more likely to become functionally dependent or be admitted to nursing homes in the months following hospital discharge.^33^, ^34^

The lack of validation cohort is a limitation of our study, along with its retrospective character. However, this is the first worldwide report on predictors of late mortality, results are definite, sample size is adequate because of large number of events, and documentation is complete. Moreover, to increase the robustness of our results (internal validation), we employed resampling techniques and calculated normal‐based confidence intervals after bootstrapping with 1000 replicates for (i) coefficients of 4C and CFS in multivariable (including age, sex, and eGFR) logistic regression models of early and late mortality and (ii) difference in AUC for 4C and CFS with respect to late mortality, which validated our results. Thus, these results at the least consist a first proof of concept that premorbid status is cardinal for late mortality, strongly supporting the concept of recognition of frail patients at risk for mortality due to COVID‐19, and encouraging the application of strategies of aggressive preventive measures in these patients such as booster vaccination and social distancing.

In conclusion, late mortality in our cohort of patients hospitalized with COVID‐19 was associated with baseline frailty status, and not with measures of severity of the acute infection phase, in contrast with early mortality. We suggest that clinically frail patients are at especially high risk of poor long‐term outcomes and represent a key vulnerable group requiring close medical attention after hospital discharge.

## Conflict of interest

None declared.

## Funding

K.F.B. is funded by a National Institute for Health Research (NIHR) Clinical Lectureship (CL‐2017‐01‐004). A.T.H. is funded by an NIHR Academic Clinical Fellowship (ACF‐2018‐01‐004) and the British Medical Association Foundation. C.J.A.D. is funded by the Wellcome Trust (211153/Z/18/Z). K. Stellos is supported by grants from the European Research Council (ERC) under the European Union's Horizon 2020 research and innovation programme (MODVASC, Grant Agreement No. 759248) and the German Research Foundation [Deutsche Forschungsgemeinschaft (DFG); SFB834 B12, Project Number 75732319].

## Supporting information


**Table S1.** Description of the Clinical Frailty Scale.
**Table S2.** Description of the 4C Mortality Score.
**Table S3.** Measures of explained variation for different sets of confounders in the final multivariable model.
**Table S4.** Additive value of CFS over the core models for 28‐day and late mortality.
**Table S5.** Cross validation of AUC for the main multivariable models.
**Table S6.** Univariate associations of clinical characteristics with early (28‐day) and late mortality.
**Table S7.** Univariate associations of laboratory parameters with early (28‐day) and late mortality.
**Table S8.** Association of disease features with early (28‐day) and late mortality after adjustment for age, sex and eGFR.
**Table S9.** Clinical and laboratory features as predictors of total 9‐month long‐term COVID‐19 mortality (combined early and late mortality).
**Table S10.** Multivariable analysis of predictors for total 9‐month long‐term mortality (combined early and late mortality).
**Table S11.** Multivariable analysis of predictors for 28‐day mortality using a cutoff of CFS ≥ 5.
**Table S12.** Multivariable analysis of predictors for late mortality using a cutoff of CFS ≥ 5.
**Table S13.** Association of disease features with early (28‐day) and late mortality adjusted for Clinical Frailty Scale.
**Table S14.** Association of Clinical Frailty Scale with early (28‐day) and late mortality according to age‐group.
**Figure S1.** Smoothed restricted cubic spline plots of the odds ratio (OR) for overall mortality in patients with COVID‐19 versus the baseline levels of A. Clinical Frailty Scale (CFS) and B. 4C mortality score. Depicted odds ratios indicate comparisons to the reference point (median value of CFS or 4C in our cohort). To enhance visual clarity, graphs were limited to the 95th percentile of corresponding scores. Three knots were fixed at the 10th, 50th, and 90th percentile of CFS and 4C distribution. The upper‐ and lower‐most dashed curves represent the 95% CI of the predicted ORs (middle solid line).
**Figure S2.** Receiver operating characteristic (ROC) curves and corresponding area(s) under the curve (AUC) for total 9‐month mortality in patients hospitalized with COVID‐19. The discriminatory performance of the 4C Mortality and the Clinical Frailty Score for the occurrence of death is graphically assessed by corresponding ROC curves and AUCs are compared to derive P‐values using the chi‐square distribution with one degree of freedom.

## References

[1] World Health Organization. WHO coronavirus (COVID‐19) dashboard. https://covid19.who.int/ (Last accessed: 15 February 2022).

[2] Knight SR , Ho A , Pius R , Buchan I , Carson G , Drake TM , et al. Risk stratification of patients admitted to hospital with COVID‐19 using the ISARIC WHO Clinical Characterisation Protocol: development and validation of the 4C Mortality Score. BMJ 2020;370:m3339. 32907855 10.1136/bmj.m3339 PMC7116472

[3] Huang C , Huang L , Wang Y , Li X , Ren L , Gu X , et al. 6‐month consequences of COVID‐19 in patients discharged from hospital: a cohort study. Lancet 2021;397:220–232. 33428867 10.1016/S0140-6736(20)32656-8 PMC7833295

[4] Ayoubkhani D , Khunti K , Nafilyan V , Maddox T , Humberstone B , Diamond I , et al. Post‐COVID syndrome in individuals admitted to hospital with COVID‐19: retrospective cohort study. BMJ 2021;372:n693. 33789877 10.1136/bmj.n693 PMC8010267

[5] Giannis D , Allen SL , Tsang J , Flint S , Pinhasov T , Williams S , et al. Postdischarge thromboembolic outcomes and mortality of hospitalized patients with COVID‐19: the CORE‐19 registry. Blood 2021;137:2838–2847. 33824972 10.1182/blood.2020010529 PMC8032474

[6] Baker KF , Hanrath AT , van der Loeff IS , Tee S , Capstick R , Marchitelli G , et al. COVID‐19 management in a UK NHS Foundation Trust with a high consequence infectious diseases centre: a retrospective analysis. Med Sci (Basel) 2021;9:6. 33557238 10.3390/medsci9010006 PMC7931073

[7] Rockwood K , Song X , MacKnight C , Bergman H , Hogan DB , McDowell I , et al. A global clinical measure of fitness and frailty in elderly people. CMAJ 2005;173:489–495. 16129869 10.1503/cmaj.050051 PMC1188185

[8] Rockwood K , Theou O . Using the Clinical Frailty Scale in allocating scarce health care resources. Can Geriatr J 2020;23:210–215. 32904824 10.5770/cgj.23.463 PMC7458601

[9] Sablerolles RSG , Lafeber M , van Kempen JAL , van de Loo BPA , Boersma E , Rietdijk WJR , et al. Association between Clinical Frailty Scale score and hospital mortality in adult patients with COVID‐19 (COMET): an international, multicentre, retrospective, observational cohort study. Lancet Healthy Longev 2021;2:e163–e170. 33655235 10.1016/S2666-7568(21)00006-4 PMC7906710

[10] Charlson ME , Pompei P , Ales KL , MacKenzie CR . A new method of classifying prognostic comorbidity in longitudinal studies: development and validation. J Chronic Dis 1987;40:373–383. 3558716 10.1016/0021-9681(87)90171-8

[11] Gönen M . Analyzing Receiver Operating Characteristic Curves with SAS. SAS Institute Inc; 2007.

[12] Heinzl H , Waldhör T , Mittlböck M . Careful use of pseudo *R*‐squared measures in epidemiological studies. Stat Med 2005;24:2867–2872. 16134131 10.1002/sim.2168

[13] Bozdogan H . Model selection and Akaike's information criterion (AIC): the general theory and its analytical extensions. Psychometrika 1987;52:345–370.

[14] Akaike H . Likelihood of a model and information criteria. J Econom 1981;16:3–14.

[15] Akaike H . Information measures and model selection. Int Stat Inst 1983;44:277–291.

[16] Smith GCS , Seaman SR , Wood AM , Royston P , White IR . Correcting for optimistic prediction in small data sets. Am J Epidemiol 2014;180:318–324. 24966219 10.1093/aje/kwu140 PMC4108045

[17] Aliberti MJR , Szlejf C , Avelino‐Silva VI , Suemoto CK , Apolinario D , Dias MB , et al. COVID‐19 is not over and age is not enough: using frailty for prognostication in hospitalized patients. J Am Geriatr Soc 2021;69:1116–1127. 33818759 10.1111/jgs.17146 PMC8251205

[18] Zhou F , Yu T , Du R , Fan G , Liu Y , Liu Z , et al. Clinical course and risk factors for mortality of adult inpatients with COVID‐19 in Wuhan, China: a retrospective cohort study. Lancet 2020;395:1054–1062. 32171076 10.1016/S0140-6736(20)30566-3 PMC7270627

[19] Lazaridis C , Vlachogiannis NI , Bakogiannis C , Spyridopoulos I , Stamatelopoulos K , Kanakakis I , et al. Involvement of cardiovascular system as the critical point in coronavirus disease 2019 (COVID‐19) prognosis and recovery. Hellenic J Cardiol 2020;61:381–395. 32534109 10.1016/j.hjc.2020.05.004 PMC7286275

[20] Wu Z , McGoogan JM . Characteristics of and important lessons from the coronavirus disease 2019 (COVID‐19) outbreak in China: summary of a report of 72 314 cases from the Chinese center for disease control and prevention. JAMA 2020;323:1239–1242. 32091533 10.1001/jama.2020.2648

[21] Petermann‐Rocha F , Pell JP , Celis‐Morales C , Ho FK . Frailty, sarcopenia, cachexia and malnutrition as comorbid conditions and their associations with mortality: a prospective study from UK Biobank. J Public Health 2021;fdaa226. 10.1093/pubmed/fdaa226 PMC9234318 33423060

[22] Kanenawa K , Isotani A , Yamaji K , Nakamura M , Tanaka Y , Hirose‐Inui K , et al. The impact of frailty according to Clinical Frailty Scale on clinical outcome in patients with heart failure. ESC Heart Fail 2021;8:1552–1561. 33547759 10.1002/ehf2.13254 PMC8006666

[23] Zhang X‐M , Jiao J , Cao J , Huo XP , Zhu C , Wu XJ , et al. Frailty as a predictor of mortality among patients with COVID‐19: a systematic review and meta‐analysis. BMC Geriatr 2021;21:186. 33731018 10.1186/s12877-021-02138-5 PMC7968577

[24] Blomaard LC , van der Linden CMJ , van der Bol JM , Jansen SWM , Polinder‐Bos HA , Willems HC , et al. Frailty is associated with in‐hospital mortality in older hospitalised COVID‐19 patients in the Netherlands: the COVID‐OLD study. Age Ageing 2021;50:631–640. 33951156 10.1093/ageing/afab018 PMC7929372

[25] Hewitt J , Carter B , Vilches‐Moraga A , Quinn TJ , Braude P , Verduri A , et al. The effect of frailty on survival in patients with COVID‐19 (COPE): a multicentre, European, observational cohort study. Lancet Public Health 2020;5:e444–e451. 32619408 10.1016/S2468-2667(20)30146-8 PMC7326416

[26] Geriatric Medicine Research Collaborative , Covid Collaborative , Welch C . Age and frailty are independently associated with increased COVID‐19 mortality and increased care needs in survivors: results of an international multi‐centre study. Age Ageing 2021;50:617–630. 33543243 10.1093/ageing/afab026 PMC7929433

[27] Vilches‐Moraga A , Price A , Braude P , Pearce L , Short R , Verduri A , et al. Increased care at discharge from COVID‐19: the association between pre‐admission frailty and increased care needs after hospital discharge; a multicentre European observational cohort study. BMC Med 2020;18:408. 33334341 10.1186/s12916-020-01856-8 PMC7746415

[28] Lambden S , Laterre PF , Levy MM , Francois B . The SOFA score—development, utility and challenges of accurate assessment in clinical trials. Crit Care 2019;23:374. 31775846 10.1186/s13054-019-2663-7 PMC6880479

[29] Rockwood K . Rationing care in COVID‐19: if we must do it, can we do better? Age Ageing 2021;50:3–6. 32939534 10.1093/ageing/afaa202 PMC7543265

[30] Gulliford MC , Charlton J , Winter JR , Sun X , Rezel‐Potts E , Bunce C , et al. Probability of sepsis after infection consultations in primary care in the United Kingdom in 2002–2017: Population‐based cohort study and decision analytic model. PLoS Med. 2020;17:e1003202. 32702001 10.1371/journal.pmed.1003202 PMC7377386

[31] Fernando SM , McIsaac DI , Perry JJ , Rochwerg B , Bagshaw SM , Thavorn K , et al. Frailty and associated outcomes and resource utilization among older ICU patients with suspected infection. Crit Care Med 2019;47:e669–e676. 31135504 10.1097/CCM.0000000000003831

[32] Prescott HC , Osterholzer JJ , Langa KM , Angus DC , Iwashyna TJ . Late mortality after sepsis: propensity matched cohort study. BMJ 2016;353:i2375. 27189000 10.1136/bmj.i2375 PMC4869794

[33] Ferrante LE , Pisani MA , Murphy TE , Gahbauer EA , Leo‐Summers LS , Gill TM . The association of frailty with post‐ICU disability, nursing home admission, and mortality: a longitudinal study. Chest 2018;153:1378–1386. 29559308 10.1016/j.chest.2018.03.007 PMC6026287

[34] Bagshaw SM , Stelfox HT , McDermid RC , Rolfson DB , Tsuyuki RT , Baig N , et al. Association between frailty and short‐ and long‐term outcomes among critically ill patients: a multicentre prospective cohort study. CMAJ 2014;186:E95–E102. 24277703 10.1503/cmaj.130639 PMC3903764

[35] Hope AA , Law J , Nair R , Kim M , Verghese J , Gong MN . Frailty, acute organ dysfunction, and increased disability after hospitalization in older adults who survive critical illness: a prospective cohort study. J Intensive Care Med 2020;35:1505–1512. 31607212 10.1177/0885066619881115

[36] von Haehling S , Morley JE , Coats AJS , Anker SD . Ethical guidelines for publishing in the Journal of Cachexia, Sarcopenia and Muscle: update 2021. J Cachexia Sarcopenia Muscle 2021;12:2259–2261. 34904399 10.1002/jcsm.12899 PMC8718061

