# Supplementary material for: Clinical frailty, and not features of acute infection, is associated with late mortality in COVID‐19: a retrospective cohort study
Source: J Cachexia Sarcopenia Muscle. 2022 Mar 7;13(3):1502–13. doi: 10.1002/jcsm.12966 (PMC9088314; doi:10.1002/jcsm.12966)
Supplement: Supplementary file 1 — Table S1. Description of the Clinical Frailty Scale. Table S2. Description of the 4C Mortality Score. Table S3. Measures of explained variation for different sets of confounders in the final multivariable model. Table S4. Additive value of CFS over the core models for 28‐day and late mortality. Table S5. Cross validation of AUC for the main multivariable models. Table S6. Univariate associations of clinical characteristics with early (28‐day) and late mortality. Table S7. Univariate associations of laboratory parameters with early (28‐day) and late mortality. Table S8. Association of disease features with early (28‐day) and late mortality after adjustment for age, sex and eGFR. Table S9. Clinical and laboratory features as predictors of total 9‐month long‐term COVID‐19 mortality (combined early and late mortality). Table S10. Multivariable analysis of predictors for total 9‐month long‐term mortality (combined early and late mortality). Table S11. Multivariable analysis of predictors for 28‐day mortality using a cutoff of CFS ≥ 5. Table S12. Multivariable analysis of predictors for late mortality using a cutoff of CFS ≥ 5. Table S13. Association of disease features with early (28‐day) and late mortality adjusted for Clinical Frailty Scale. Table S14. Association of Clinical Frailty Scale with early (28‐day) and late mortality according to age‐group. Figure S1. Smoothed restricted cubic spline plots of the odds ratio (OR) for overall mortality in patients with COVID‐19 versus the baseline levels of A. Clinical Frailty Scale (CFS) and B. 4C mortality score. Depicted odds ratios indicate comparisons to the reference point (median value of CFS or 4C in our cohort). To enhance visual clarity, graphs were limited to the 95th percentile of corresponding scores. Three knots were fixed at the 10th, 50th, and 90th percentile of CFS and 4C distribution. The upper‐ and lower‐most dashed curves represent the 95% CI of the predicted ORs (middle solid line). Figure S2. Receiver ope [file JCSM-13-1502-s001.pdf]

Supplementary material for

Clinical frailty, and not features of acute infection, is associated with late mortality in COVID-19: a retrospective cohort study

Nikolaos I. Vlachogiannis[1,2,*], Kenneth F. Baker[2,3,4,*], Georgios Georgiopoulos[1,5,*], Charalampos Lazaridis[2] , Ina Schim van der Loeff[3], Aidan T. Hanrath[3], Kateryna Sopova[2], Simon Tual-Chalot[1], Aikaterini Gatsiou[1], Ioakim Spyridopoulos[2-4], Kimon Stamatelopoulos[1,5,*], Christopher J. A. Duncan[2-4,*], Konstantinos Stellos[1,2,4,6,7*]

[1]Biosciences Institute, Vascular Biology and Medicine Theme, Faculty of Medical Sciences, Newcastle University, Newcastle Upon Tyne, UK.

[2]RVI and Freeman Hospitals, Newcastle upon Tyne Hospitals NHS Foundation Trust, Newcastle Upon Tyne, UK.

[3]Translational and Clinical Research Institute, Newcastle University, Newcastle Upon Tyne, UK.

[4]NIHR Newcastle Biomedical Research Centre, Newcastle University and Newcastle upon Tyne Hospitals NHS Foundation Trust

[5]Department of Clinical Therapeutics, National and Kapodistrian University of Athens Medical School, Athens, Greece.

[6]Department of Cardiovascular Research, European Center for Angioscience (ECAS), Heidelberg University, Mannheim, Germany

[7]German Centre for Cardiovascular Research (DZHK), partner site Heidelberg/Mannheim, Mannheim, Germany

* Equal first/senior authorship

Correspondence:

Kenneth F. Baker, PhD MRCP; Translational and Clinical Research Institute, Newcastle University, Newcastle Upon Tyne NE2 4HH, UK; Email: kenneth.baker@newcastle.ac.uk

or

Konstantinos Stellos, MD; Biosciences Institute, Newcastle University, International Centre for Life, Central Parkway, Newcastle upon Tyne, NE1 3BZ, UK; Email: konstantinos.stellos@newcastle.ac.uk

Table S1. Description of the Clinical Frailty Scale*

1	Very fit	Among the fittest people for their age.
2	Fit	Fit, occasionally active exercise.
3	Managing well	Well controlled medical problems, not regularly active.
4	Living with very mild frailty	Independent, but often symptoms limit their activities.
5	Living with mild frailty	Needing help with high order instrumental activities of everyday life.
6	Living with moderate frailty	Needing help with all outdoor activities and with housekeeping.
7	Living with severe frailty	Completely dependent on others for personal care.
8	Living with very severe frailty	Approaching end of life.
9	Terminally ill	People with <6 months life expectancy due to underlying conditions, but who do not have severe frailty.

*Adapted from *Can Geriatr J. 2020 Sep 1;23(3):210-215.*

**Table S2. Description of the 4C Mortality Score***	
**Variable**	**Variable value (4C mortality scoring)**
Age (years)	<50 **(0)**<br>50-59 **(2)**<br>60-69 **(4)**<br>70-79 **(6)**<br>≥80 **(7)**
Sex	Female **(0)**<br>Male **(1)**
Number of comorbidities according to Charlson comorbidity index & physician-assessed obesity	0 **(0)**<br>1 **(1)**<br>≥2 **(2)**
Respiratory rate (breaths/minute)	<20 **(0)**<br>20-29 **(1)**<br>≥30 **(2)**
Peripheral oxygen saturation (%) on room air	≥92 **(0)**<br><92 **(2)**
Glasgow Coma Scale	15 **(0)**<br>≤15 **(2)**
Urea (mmoL/L)	<7 **(0)**<br>7-14 **(1)**<br>>14 **(3)**
C-reactive protein (mg/L)	<50 **(0)**<br>50-99 **(1)**<br>≥100 **(2)**
	Range: **0-21**
*Adapted from *BMJ. 2020 Sep 9;370:m3339*.	

Table S3: Measures of explained variation for different sets of confounders in the final multivariable model

	**Adjusted R squared index (%)***
**In hospital**	
Core model (clinical)	18.6
Core model (clinical)+CFS	21.3
Core model (laboratory)	15.6
Core model (laboratory)+CFS	19.9
**Late mortality**	
Core model (clinical)	12.6
Core model (clinical)+CFS	23.4
Core model (laboratory)	7.1
Core model (laboratory)+CFS	21.6

*Adjusted R squared index is a measure of explained variation. Higher R squared index indicates better model fit to observed data.

Clinical model for 28-day mortality included age>65 years, shortness of breath, myalgia, confusion, respiratory rate>20 (breaths/min.), hypoxia (SpO2<92%), congestive heart failure, chronic kidney disease, hypertension and dementia.

Laboratory model for 28-day mortality included white blood cells (WBCs)<4 or WBCs>12 (x10^9/L), neutrophil number, urea, estimated glomerular filtration rate (eGFR), alkaline phosphatase (ALP), C-reactive protein (CRP), albumin and sodium.

Clinical model for late mortality included age>65 years, confusion, active cancer, ischemic heart disease and dementia.

Laboratory model for late mortality included haemoglobin, neutrophil number, estimated glomerular filtration rate (eGFR), alkaline phosphatase (ALP) and albumin.

Abbreviations: CFS: clinical frailty scale.

Table S4. Additive value of CFS over the core models for 28-day and late mortality		
	**AIC**	**P-value**
**28-day mortality**		
**Clinical model**	437.1	
Clinical model + CFS	424.9	<0.001
Clinical model + CFS≥5	429.1	0.002
**Laboratory model**	418.1	
Laboratory model + CFS	395.3	<0.001
Laboratory model + CFS≥5	400.5	<0.001
**Late mortality**		
**Clinical model**	220.0	
Clinical model + CFS	191.6	<0.001
Clinical model + CFS≥5	200.0	<0.001
**Laboratory model**	206.5	
Laboratory model + CFS	173.1	<0.001
Laboratory model + CFS ≥5	179.2	<0.001

Clinical model for 28-day mortality included age>65 years, shortness of breath, myalgia, confusion, respiratory rate>20 (breaths/min.), hypoxia (SpO2<92%), congestive heart failure, chronic kidney disease, hypertension and dementia.
Laboratory model for 28-day mortality included white blood cells (WBCs)<4 or WBCs>12 (x10^9/L), neutrophil number, urea, estimated glomerular filtration rate (eGFR), alkaline phosphatase (ALP), C-reactive protein (CRP), albumin and sodium.
Clinical model for late mortality included age>65 years, confusion, active cancer, ischemic heart disease and dementia.
Laboratory model for late mortality included haemoglobin, neutrophil number, estimated glomerular filtration rate (eGFR), alkaline phosphatase (ALP) and albumin.
Abbreviations: AIC: Akaike information criterion; CFS: clinical frailty scale.

Table S5. Cross validation of AUC for the main multivariable models		
	**Training set (50%)**	**Validation set (50%)**
	**AUC (95% CI)**	**AUC (95% CI)**
**28-day mortality**		
Clinical model	0.795 (0.791-0.798)	0.795 (0.790-0.796)
Laboratory model	0.784 (0.764-0.794)	0.783 (0.768-0.794)
**Late mortality**		
Clinical model	0.710 (0.638-0.748)	0.719 (0.604-0.768)
Laboratory model	0.843 (0.779-0.861)	0.893 (0.802-0.864)

Clinical model for 28-day mortality included age>65 years, shortness of breath, myalgia, confusion, respiratory rate>20 (breaths/min.), hypoxia (SpO_2<92%), congestive heart failure, chronic kidney disease, hypertension and dementia.

Laboratory model for 28-day mortality included white blood cells (WBCs)<4 or WBCs>12 (x10^9/L), neutrophil number, urea, estimated glomerular filtration rate (eGFR), alkaline phosphatase (ALP), C-reactive protein (CRP), albumin and sodium.

Clinical model for late mortality included age>65 years, confusion, active cancer, ischemic heart disease and dementia.

Laboratory model for late mortality included haemoglobin, neutrophil number, estimated glomerular filtration rate (eGFR), alkaline phosphatase (ALP) and albumin.

Note: Close concordance in AUCs of the two datasets (training and validation) indicates absence of overfitting.

Abbreviations: AUC: area under the curve; CI: confidence interval.

Table S6. Univariate associations of clinical characteristics with early (28-day) and late mortality						
	Early mortality			Late mortality		
	**Survived (n=351)**	**Died (n=120)**	**P-value***	**Survived (n=310)**	**Died (n=41)**	**P-value***
**Demographics**						
Age (years)	**69 (24)**	**82 (15)**	**<0.001**	**66 (24)**	**83 (18)**	**<0.001**
Male sex	181 (51.6)	67 (55.8)	0.459	162 (52.3)	19 (46.3)	0.509
**Symptoms and signs at presentation**						
T>38°C or T<36°C	82 (24.5)	31 (26.5)	0.710	71 (24.1)	11 (26.8)	0.701
Cough	237 (68.5)	76 (63.3)	0.311	211 (69.2)	26 (63.4)	0.476
Sputum	85 (24.6)	21 (17.5)	0.130	76 (24.9)	9 (22.0)	0.847
Shortness of breath	201 (58.1)	82 (68.3)	0.051	180 (59.0)	21 (51.2)	0.400
Haemoptysis	11 (3.2)	0 (0)	0.074	10 (3.3)	1 (2.4)	1.000
Rhinorrhea	15 (4.3)	2 (1.7)	0.260	13 (4.3)	2 (4.9)	0.694
Pharyngitis	**36 (10.4)**	**0 (0)**	**<0.001**	34 (11.1)	2 (4.9)	0.284
Myalgia	**74 (21.4)**	**11 (9.2)**	**0.002**	70 (23.0)	4 (9.8)	0.066
Arthralgia	9 (2.6)	0 (0)	0.120	8 (2.6)	1 (2.4)	1.000
Fatigue	132 (38.2)	46 (38.3)	1.000	117 (38.4)	15 (36.6)	0.866
Headache	37 (10.7)	8 (6.7)	0.281	**37 (12.1)**	**0 (0)**	**0.013**
Confusion	**69 (19.9)**	**48 (40.0)**	**<0.001**	**54 (17.7)**	**15 (36.6)**	**0.011**
Anosmia	21 (6.1)	4 (3.3)	0.348	19 (6.2)	2 (4.9)	1.000
Abdominal pain	44 (12.7)	11 (9.2)	0.330	40 (13.1)	4 (9.8)	0.802
Diarrhea	69 (19.9)	16 (13.3)	0.131	63 (20.7)	6 (14.6)	0.532
Heart rate (bpm)	89 (28)	90 (31)	0.415	90 (29)	85 (24)	0.272
Heart rate > 90 bpm	154 (46.1)	57 (49.1)	0.591	141 (48.0)	13 (32.5)	0.090
Systolic BP (mmHg)	126 (33)	126 (27)	0.975	126 (33)	124 (36)	0.806
Diastolic BP (mmHg)	73 (17)	70 (14)	0.068	**74 (17)**	**70 (19)**	**0.039**
RR (breaths/min)	**20 (6)**	**22 (10)**	**<0.001**	20 (6)	20 (6)	0.780
RR > 20 (breaths/min)	**144 (43.2)**	**71 (61.2)**	**0.001**	126 (43.0)	18 (45.0)	0.866
Hypoxia (SpO2<92%)	**134 (40.1)**	**70 (59.8)**	**<0.001**	116 (39.6)	18 (43.9)	0.613
Mean BP (mmHg)	94 (20)	94 (19)	0.362	95 (20)	92 (26)	0.365
**Co-morbidities**						
Active cancer	37 (10.6)	19 (16.0)	0.140	**27 (8.8)**	**10 (24.4)**	**0.005**
Asthma	45 (12.9)	10 (8.3)	0.249	42 (13.6)	3 (7.3)	0.328
COPD	52 (14.9)	19 (15.8)	0.883	42 (13.6)	10 (24.4)	0.098
Other ILD	16 (4.6)	7 (5.8)	0.625	14 (4.6)	2 (4.9)	1.000
Ischemic heart disease	67 (19.2)	28 (23.3)	0.357	**52 (16.9)**	**15 (36.6)**	**0.005**
Congestive heart failure	**37 (10.6)**	**31 (25.8)**	**<0.001**	30 (9.8)	7 (17.1)	0.175
Chronic kidney disease	**72 (20.6)**	**41 (34.2)**	**0.004**	60 (19.5)	12 (29.3)	0.153
DM diet-controlled	31 (8.9)	15 (12.5)	0.285	28 (9.1)	3 (7.3)	1.000
DM tabs-controlled	37 (10.6)	14 (11.7)	0.736	31 (10.1)	6 (14.6)	0.415
DM- insulin	21 (6.0)	8 (6.7)	0.826	19 (6.2)	2 (4.9)	1.000
Hypertension	**124 (35.5)**	**65 (54.2)**	**0.001**	108 (35.1)	16 (39.0)	0.607
Liver disease	19 (5.4)	2 (1.7)	0.122	16 (5.2)	3 (7.3)	0.477
Dementia	**51 (14.6)**	**37 (30.8)**	**<0.001**	**33 (10.7)**	**18 (43.9)**	**<0.001**
WHO ordinal scale at admission (>4)	**156 (44.8)**	**36 (30.0)**	**0.005**	139 (45.3)	17 (41.5)	0.739
WHO ordinal scale at discharge (>4)	**335 (98.8)**	**0 (0)**	**<0.001**	297 (99.3)	38 (95.0)	0.070

Continuous variables are presented as median (inter-quartile range) and categorical variables as absolute count (valid percentage).

*P-values are derived from Mann Whitney U test for continuous variables and Fisher's exact test for categorical variables.

Abbreviations: T: temperature; mmHg: millimeters of mercury; bpm: beats per minute; BP: blood pressure; RR: respiratory rate; COPD: chronic obstructive pulmonary disease; ILD: interstitial lung disease; DM: diabetes mellitus; WHO: World Health Organization.

Table S7. Univariate associations of laboratory parameters with early (28-day) and late mortality

	Early mortality			Late mortality		
	**Survived (n=351)**	**Died (n=120)**	**P-value***	**Survived (n=310)**	**Died (n=41)**	**P-value***
**Laboratory parameters**						
Haemoglobin (g/L)	131 (31)	128 (33)	0.242	**133 (27)**	**113 (34)**	**<0.001**
WBCs (x10^9/L)	**7.3 (4)**	**8.1 (6)**	**0.003**	**7.1 (4)**	**7.9 (4)**	**0.015**
WBCs<4 or WBCs>12 (x10^9/L)	**58 (17.0)**	**35 (29.7)**	**0.005**	50 (16.6)	8 (19.5)	0.658
Neutrophils (x10^9/L)	**5.5 (3)**	**6.6 (5)**	**<0.001**	5.3 (3)	5.9 (4)	0.067
Eosinophils (x10^9/L)	0.01 (0)	0.01 (0)	0.073	0.01 (0)	0.01 (0)	0.401
Lymphocytes (x10^9/L)	**0.96 (0.7)**	**0.85 (0.8)**	**0.027**	0.96 (0.7)	0.97 (0.8)	0.818
Platelets (x10^9/L)	222 (112)	223 (122)	0.959	217 (109)	246 (166)	0.254
Urea (mmol/L)	**6.5 (6)**	**9.8 (10)**	**<0.001**	**6.3 (5)**	**8.3 (6)**	**0.027**
Creatinine (μmol/L)	**81 (39)**	**104 (72)**	**<0.001**	81 (37)	82 (54)	0.605
eGFR (mL/min/1.73 m^2)	**76 (39)**	**49 (44)**	**<0.001**	**77 (37)**	**59 (38)**	**0.021**
ALP (IU/L)	**79 (41)**	**88 (45)**	**0.003**	**78 (37)**	**98 (103)**	**0.013**
Bilirubin (μmol/L)	8 (5)	8 (7)	0.453	8 (5)	7.5 (6)	0.608
CRP (mg/L)	**58 (100)**	**92 (135)**	**<0.001**	57 (99)	79 (106)	0.834
Albumin (g/L)	**39 (7)**	**36 (6)**	**<0.001**	**39 (6)**	**35 (8)**	**0.001**
Sodium (mmol/L)	**137 (6)**	**138 (8)**	**0.008**	137 (6)	137 (5)	0.980
Potassium (mmol/L)	**4.1 (1)**	**4.3 (1)**	**0.047**	4.1 (1)	4.15 (1)	0.865
**Risk scores**						
Clinical Frailty Scale	**3 (4)**	**6 (3)**	**<0.001**	**3 (3)**	**6 (2)**	**<0.001**
4C mortality score	**10 (6)**	**14 (4)**	**<0.001**	**9 (6)**	**12.5 (4)**	**<0.001**

Continuous variables are presented as median (inter-quartile range) and categorical variables as absolute count (valid percentage).
*P-values are derived from Mann Whitney U test for continuous variables and Fisher's exact test for categorical variables.
Abbreviations: WBCs; white blood cells; eGFR: estimated glomerular filtration rate; ALP: alkaline phosphatase; CRP: C-reactive protein.

Table S8. Association of disease features with early (28-day) and late mortality after adjustment for age, sex and eGFR

	**Early mortality**		**Late mortality**	
	**OR (95% CI)**	**P-value***	**OR (95% CI)**	**P-value***
**Symptoms and signs at presentation**				
Shortness of breath	**2.23 (1.35-3.66)**	**0.002**	0.92 (0.46-1.85)	0.825
Myalgia	0.79 (0.38-1.67)	0.545	0.83 (0.26-2.61)	0.747
Confusion	1.23 (0.74-2.06)	0.428	1.28 (0.59-2.77)	0.538
Respiratory rate (breaths/min.)	**1.08 (1.04-1.13)**	**<0.001**	1.02 (0.95-1.08)	0.646
RR>20 (breaths/min.)	**2.51 (1.54-4.08)**	**<0.001**	1.24 (0.62-2.49)	0.539
Hypoxia (SpO2<92%)	**2.49 (1.54-4.04)**	**<0.001**	1.29 (0.64-2.57)	0.478
**Co-morbidities**				
Active Cancer	1.56 (0.81-3.00)	0.186	**3.18 (1.33-7.60)**	**0.009**
Ischemic heart disease	0.79 (0.46-1.36)	0.396	1.79 (0.86-3.75)	0.122
Congestive heart failure	**1.94 (1.09-3.43)**	**0.023**	1.23 (0.48-3.12)	0.666
Chronic kidney disease	0.70 (0.38-1.27)	0.237	0.85 (0.33-2.21)	0.747
Hypertension	1.35 (0.85-2.14)	0.210	0.71 (0.35-1.44)	0.342
Dementia	1.09 (0.63-1.91)	0.749	**2.95 (1.30-6.70)**	**0.010**
Adapted WHO ordinal scale at admission	**0.50 (0.37-0.69)**	**<0.001**	0.91 (0.56-1.49)	0.714
**Laboratory parameters**				
Haemoglobin (g/L)	0.999 (0.988-1.009)	0.826	**0.975 (0.959-0.992)**	**0.004**
WBCs<4 or WBCs>12 (x10^9/L)	**1.92 (1.12-3.27)**	**0.017**	1.20 (0.50-2.85)	0.687
Neutrophils (x10^9/L)	**1.12 (1.05-1.20)**	**<0.001**	1.10 (0.99-1.22)	0.063
Urea (mmol/L)	1.02 (0.98-1.06)	0.321	0.99 (0.92-1.06)	0.762
ALP (IU/L)	1.002 (1.000-1.005)	0.076	**1.006 (1.002-1.010)**	**0.005**
CRP (mg/L)	**1.005 (1.003-1.008)**	**<0.001**	0.999 (0.995-1.004)	0.764
Albumin (g/L)	**0.94 (0.90-0.99)**	**0.015**	**0.92 (0.86-0.99)**	**0.020**
Sodium (mmol/L)	1.035 (0.998-1.073)	0.060	0.97 (0.90-1.03)	0.305
**Risk scores**				
Clinical Frailty Scale	**1.25 (1.08-1.44)**	**0.003**	**1.96 (1.51-2.53)**	**<0.001**
4C mortality score	**1.25 (1.14-1.38)**	**<0.001**	1.14 (0.99-1.31)	0.066

*Odds ratios are derived from binary logistic regression with early (28-day) mortality or late mortality as the dependent variable, and per one unit increase for continuous variables, or versus the reference category for categorical variables plus age, sex and eGFR as independent variables.

Abbreviations: eGFR: estimated glomerular filtration rate; OR: odds ratio; CI: confidence intervals; RR: respiratory rate; WHO: World Health Organization; WBCs: white blood cells; ALP: alkaline phosphatase, CRP: C-reactive protein.

**Table S9. Clinical and laboratory features as predictors of total 9-month long-term COVID-19 mortality (combined early and late mortality)**		
	**OR (95% CI)***	**P-value**
**Demographics**		
Age (years)	**1.08 (1.06-1.10)**	**<0.001**
Age> 65 years	**7.01 (4.13-11.88)**	**<0.001**
Male sex	1.05 (0.72-1.53)	0.811
**Symptoms and signs at presentation**		
T>38°C or T<36°C	1.14 (0.73-1.77)	0.569
Cough	0.77 (0.52-1.15)	0.203
Sputum	0.69 (0.43-1.11)	0.125
Shortness of breath	1.23 (0.83-1.83)	0.298
Haemoptysis	0.18 (0.02-1.45)	0.108
Rhinorrhea	0.57 (0.18-1.78)	0.336
Pharyngitis	**0.10 (0.02-0.42)**	**0.002**
Myalgia	**0.34 (0.19-0.63)**	**<0.001**
Arthralgia	0.23 (0.03-1.87)	0.170
Fatigue	0.98 (0.66-1.45)	0.920
Headache	**0.38 (0.17-0.83)**	**0.016**
Confusion	**2.99 (1.94-4.60)**	**<0.001**
Anosmia	0.58 (0.23-1.49)	0.259
Abdominal pain	0.68 (0.36-1.27)	0.229
Diarrhea	0.61 (0.36-1.03)	0.065
Heart rate (bpm)	1.00 (0.99-1.01)	0.947
Heart rate> 90 bpm	0.88 (0.60-1.30)	0.532
Systolic BP (mmHg)	1.00 (0.99-1.01)	0.727
Diastolic BP (mmHg)	0.99 (0.97-1.00)	0.061
Respiratory rate (breaths/min)	**1.05 (1.02-1.09)**	**0.001**
RR > 20 (breaths/min)	**1.76 (1.19-2.61)**	**0.005**
Hypoxia (SpO2<92%)	**1.92 (1.30-2.84)**	**0.001**
Mean BP (mmHg)	0.99 (0.98-1.01)	0.400
**Co-morbidities**		
Active Ca**ncer**	**2.30 (1.31-4.05)**	**0.004**
Asthma	0.56 (0.29-1.07)	0.079
COPD	1.39 (0.83-2.33)	0.211
Other ILD	1.24 (0.52-2.93)	0.625
Ischemic heart disease	**1.79 (1.13-2.84)**	**0.013**
Congestive heart failure	**2.85 (1.69-4.82)**	**<0.001**
Chronic kidney disease	**2.03 (1.32-3.13)**	**0.001**
DM diet-controlled	1.26 (0.67-2.35)	0.471
DM tabs-controlled	1.27 (0.70-2.30)	0.437
DM- insulin	1.01 (0.46-2.23)	0.979
Hypertension	**1.88 (1.27-2.76)**	**0.001**
Liver disease	0.58 (0.21-1.63)	0.304
Dementia	**4.32 (2.66-7.03)**	**<0.001**
Adapted WHO ordinal scale at admission	**0.70 (0.55-0.88)**	**0.003**
Adapted WHO ordinal scale at discharge	**0.28 (0.21-0.36)**	**<0.001**
**Laboratory parameters**		
Haemoglobin (g/L)	**0.986 (0.977-0.995)**	**0.002**
WBCs (x10^9/L)	1.00 (0.99-1.02)	0.772

WBCs<4 or WBCs>12 (x10^9/L)	**1.86 (1.17-2.96)**	**0.009**
Neutrophils (x10^9/L)	**1.17 (1.10-1.24)**	**<0.001**
Eosinophils (x10^9/L)	0.58 (0.04-9.09)	0.696
Lymphocytes (x10^9/L)	1.00 (1.00-1.00)	0.607
Platelets (x10^9/L)	1.00 (1.00-1.00)	0.261
Urea (mmol/L)	**1.07 (1.04-1.10)**	**<0.001**
Creatinine (µmol/L)	1.001 (0.999-1.003)	0.178
eGFR (mL/min/1.73 m^2)	**0.977 (0.970-0.985)**	**<0.001**
ALP (IU/L)	**1.005 (1.002-1.008)**	**0.001**
Bilirubin (µmol/L)	1.025 (0.999-1.053)	0.061
CRP (mg/L)	**1.003 (1.001-1.005)**	**0.005**
Albumin (g/L)	**0.90 (0.86-0.94)**	**<0.001**
Sodium (mmol/L)	**1.05 (1.01-1.08)**	**0.004**
Potassium (mmol/L)	1.25 (0.90-1.73)	0.180
**Risk scores**		
Clinical Frailty Scale	**1.72 (1.54-1.93)**	**<0.001**
4C mortality score	**1.34 (1.26-1.44)**	**<0.001**
*Odds ratios are derived from univariable binary logistic regression with total 9-month mortality as the dependent variable, and per one unit increase for continuous variables, or versus the reference category for categorical variables, as the independent variable.<br>Abbreviations: OR: odds ratio, CI: confidence intervals, T: temperature, mmHg: millimeters of mercury, bpm: beats per minute, BP: blood pressure, RR: respiratory rate, COPD: chronic obstructive pulmonary disease, ILD: interstitial lung disease, DM: diabetes mellitus, WHO: World Health Organization, WBC: white blood cells, eGFR: estimated glomerular filtration rate, ALP: alkaline phosphatase, CRP: C-reactive protein.		

Table S10. Multivariable analysis of predictors for total 9-month long-term mortality (combined early and late mortality)		
	**OR (95% C.I.)***	**P-value**
**a) Clinical characteristics**		
**CFS**	**1.57 (1.34-1.84)**	**<0.001**
**Age>65 years**	**2.27 (1.08-4.76)**	**0.030**
Pharyngitis	0.31 (0.06-1.49)	0.143
Myalgia	1.46 (0.65-3.30)	0.364
Headache	1.42 (0.52-3.86)	0.491
Confusion	1.14 (0.63-2.08)	0.659
**RR>20 (breaths/min.)**	**1.95 (1.17-3.25)**	**0.010**
Hypoxia (SpO2<92%)	1.61 (0.97-2.67)	0.065
**Active cancer**	**3.03 (1.50-6.13)**	**0.002**
Ischemic heart disease	0.97 (0.53-1.77)	0.923
Congestive heart failure	**2.13 (1.09-4.16)**	**0.028**
Chronic kidney disease	1.12 (0.66-1.91)	0.681
Hypertension	1.55 (0.94-2.56)	0.085
Dementia	1.61 (0.84-3.09)	0.156
**b) Laboratory parameters**		
**CFS**	**1.58 (1.37-1.82)**	**<0.001**
Haemoglobin	0.993 (0.980-1.005)	0.265
WBCs<4 or WBCs>12 (x10^9/L)	1.27 (0.67-2.43)	0.460
**Neutrophils (x10^9/L)**	**1.10 (1.01-1.20)**	**0.022**
Urea (mmol/L)	1.00 (0.96-1.05)	0.913
eGFR (mL/min/1.73 m^2)	0.988 (0.975-1.000)	0.058
ALP (IU/L)	1.003 (1.000-1.006)	0.073
**CRP (mg/L)**	**1.003 (1.000-1.006)**	**0.047**
Albumin (g/L)	0.97 (0.92-1.03)	0.339
Sodium (mmol/L)	1.013 (0.972-1.055)	0.551

*Odds ratios are derived from multivariable logistic regression with total 9-month long-term mortality as the dependent variable, and CFS as well as a) all clinical features, or b) all laboratory parameters associated at univariable analysis with total mortality, as independent variables.

Abbreviations: OR: odds ratio, CI: confidence intervals, CFS: clinical frailty scale, RR: respiratory rate, WBCs: white blood cells, eGFR: estimated glomerular filtration rate, ALP: alkaline phosphatase, CRP: C-reactive protein.

**Table S11. Multivariable analysis of predictors for early (28-day) mortality using a cutoff of CFS≥5**		
	**OR (95% C.I.)***	**P-value**
**a) Clinical characteristics**		
**Clinical Frailty Scale≥5**	**2.47 (1.33-4.58)**	**0.004**
**Age>65 years**	**3.94 (1.79-8.70)**	**0.001**
Shortness of breath	1.46 (0.82-2.60)	0.204
Myalgia	0.94 (0.41-2.14)	0.886
Confusion	1.63 (0.90-2.94)	0.104
**RR>20 (breaths/min.)**	**2.07 (1.23-3.50)**	**0.006**
Hypoxia (SpO2<92%)	1.65 (0.95-2.84)	0.073
**Congestive heart failure**	**2.10 (1.15-3.86)**	**0.016**
Chronic kidney disease	1.21 (0.71-2.06)	0.477
**Hypertension**	**1.71 (1.03-2.82)**	**0.036**
Dementia	1.12 (0.60-2.09)	0.732
**b) Laboratory parameters**		
**Clinical Frailty Scale≥5**	**2.70 (1.52-4.79)**	**<0.001**
WBCs<4 or WBCs>12 (x10^9/L)	1.42 (0.75-2.71)	0.286
Neutrophils (x10^9/L)	1.08 (0.999-1.17)	0.052
Urea (mmol/L)	1.01 (0.96-1.06)	0.729
**eGFR (mL/min/1.73 m^2)**	**0.985 (0.972-0.998)**	**0.023**
ALP (IU/L)	1.002 (0.999-1.005)	0.147
**CRP (mg/L)**	**1.004 (1.001-1.006)**	**0.017**
Albumin (g/L)	0.98 (0.93-1.03)	0.395
**Sodium (mmol/L)**	**1.045 (1.003-1.090)**	**0.036**

*Odds ratios are derived from multivariable logistic regression with early mortality as the dependent variable, and CFS as well as a) all clinical features, or b) all laboratory parameters associated at univariable analysis with early mortality, as independent variables. Abbreviations: OR: odds ratio, CI: confidence intervals, RR: respiratory rate, WBC: white blood cells, eGFR: estimated glomerular filtration rate, ALP: alkaline phosphatase, CRP: C-reactive protein.

Table S12. Multivariable analysis of predictors for late mortality using a cutoff of CFS≥5

	OR (95% C.I.)*	P-value
**a) Clinical characteristics**		
**Clinical Frailty Scale≥5**	**10.96 (3.50-34.27)**	**<0.001**
Age>65 years	0.91 (0.29-2.84)	0.867
Confusion	0.56 (0.21-1.51)	0.252
**Active Cancer**	**4.72 (1.75-12.76)**	**0.002**
Ischemic heart disease	1.86 (0.82-4.20)	0.136
**Dementia**	**3.82 (1.37-10.69)**	**0.011**
**b) Laboratory parameters**		
**Clinical Frailty Scale≥5**	**11.45 (3.81-34.43)**	**<0.001**
Haemoglobin (g/L)	**0.980 (0.961-0.999)**	**0.041**
Neutrophils (x10^9/L)	1.07 (0.96-1.20)	0.218
eGFR (mL/min/1.73 m^2)	0.996 (0.981-1.012)	0.643
ALP (IU/L)	1.003 (0.999-1.008)	0.156
Albumin (g/L)	0.97 (0.89-1.05)	0.420

*Odds ratios are derived from multivariable logistic regression with late mortality as the dependent variable, and CFS as well as a) all clinical features, or b) all laboratory parameters associated at univariable analysis with late mortality, as independent variables.
Abbreviations: OR: odds ratio, CI: confidence intervals, eGFR: estimated glomerular filtration rate, ALP: alkaline phosphatase.

**Table S13. Association of disease features with early (28-day) and late mortality adjusted for Clinical Frailty Scale**				
	**Early mortality**		**Late mortality**	
	**OR (95% CI)***	**P-value**	**OR (95% CI)***	**P-value**
Age (years)	**1.05 (1.03-1.07)**	**<0.001**	1.01 (0.98-1.04)	0.403
Age > 65 years	**3.29 (1.62-6.65)**	**0.001**	1.15 (0.40-3.31)	0.788
**Symptoms and signs at presentation**				
Shortness of breath	**2.30 (1.42-3.75)**	**0.001**	1.09 (0.52-2.26)	0.819
Myalgia	0.81 (0.39-1.70)	0.581	1.64 (0.47-5.73)	0.438
Confusion	1.27 (0.76-2.11)	0.367	0.79 (0.36-1.75)	0.558
RR	**1.08 (1.04-1.12)**	**<0.001**	1.01 (0.94-1.08)	0.812
Hypoxia (SpO2<92%)	**2.06 (1.30-3.26)**	**0.002**	1.00 (0.48-2.09)	0.994
Active Cancer	1.45 (0.77-2.74)	0.253	**3.77 (1.41-10.04)**	**0.008**
Ischemic heart disease	0.98 (0.58-1.67)	0.941	1.81 (0.83-3.94)	0.133
Congestive heart failure	**2.14 (1.22-3.75)**	**0.008**	0.98 (0.37-2.63)	0.968
Chronic kidney disease	1.42 (0.87-2.32)	0.156	0.99 (0.44-2.19)	0.974
Hypertension	**1.93 (1.23-3.03)**	**0.004**	1.04 (0.50-2.18)	0.918
Dementia	1.02 (0.58-1.79)	0.936	1.68 (0.75-3.76)	0.209
Adapted WHO ordinal scale at admission	**0.55 (0.41-0.75)**	**<0.001**	0.96 (0.55-1.67)	0.882
**Laboratory parameters**				
Haemoglobin (g/L)	1.002 (0.991-1.012)	0.774	**0.981 (0.964-0.998)**	**0.033**
WBCs<4 or WBCs>12 (x10^9/L)	**1.87 (1.11-3.14)**	**0.019**	0.98 (0.38-2.51)	0.961
Neutrophils (x10^9/L)	**1.11 (1.04-1.19)**	**0.001**	1.05 (0.94-1.17)	0.360
Urea (mmol/L)	**1.05 (1.02-1.08)**	**0.002**	1.01 (0.95-1.06)	0.854
eGFR (mL/min/1.73 m^2)	**0.98 (0.97-0.99)**	**<0.001**	1.00 (0.98-1.01)	0.572
ALP (IU/L)	1.002 (0.999-1.004)	0.124	1.005 (1.000-1.010)	0.070
CRP (mg/L)	**1.005 (1.003-1.008)**	**<0.001**	1.00 (0.995-1.004)	0.910
Albumin (g/L)	**0.95 (0.90-0.99)**	**0.021**	0.94 (0.87-1.01)	0.084
Sodium (mmol/L)	**1.04 (1.004-1.077)**	**0.030**	0.95 (0.89-1.01)	0.129
**Risk scores**				
4C mortality score	**1.28 (1.18-1.39)**	**<0.001**	1.06 (0.95-1.20)	0.300

*Odds ratios are derived from multivariable logistic regression with early (28-day) mortality or late mortality as the dependent variable, and per one unit increase for continuous variables, or versus the reference category for categorical variables plus Clinical Frailty Scale, as independent variables.
Abbreviations: OR: odds ratio, CI: confidence intervals, eGFR: estimated glomerular filtration rate, CRP: C-reactive protein

Table S14. Association of Clinical Frailty Scale with early (28-day) and late mortality according to age-group				
≤65 years (n=169)				
	Early mortality (n=13)		Late mortality (n=6)	
	OR (95% CI)*	P-value	OR (95% CI)*	P-value
Clinical Frailty Scale	1.42 (1.08-1.86)	0.012	2.29 (1.44-3.65)	<0.001
>65 years (n=302)				
	Early mortality (n=107)		Late mortality (n=35)	
Clinical Frailty Scale	1.31 (1.14-1.51)	<0.001	1.89 (1.42-2.53)	<0.001

*Odds ratios are derived from binary logistic regression with early (28-day) mortality or late mortality as the dependent variable, and Clinical Frailty Scale as the independent variable. Abbreviations: OR: odds ratio, CI: confidence intervals.

Figure S1

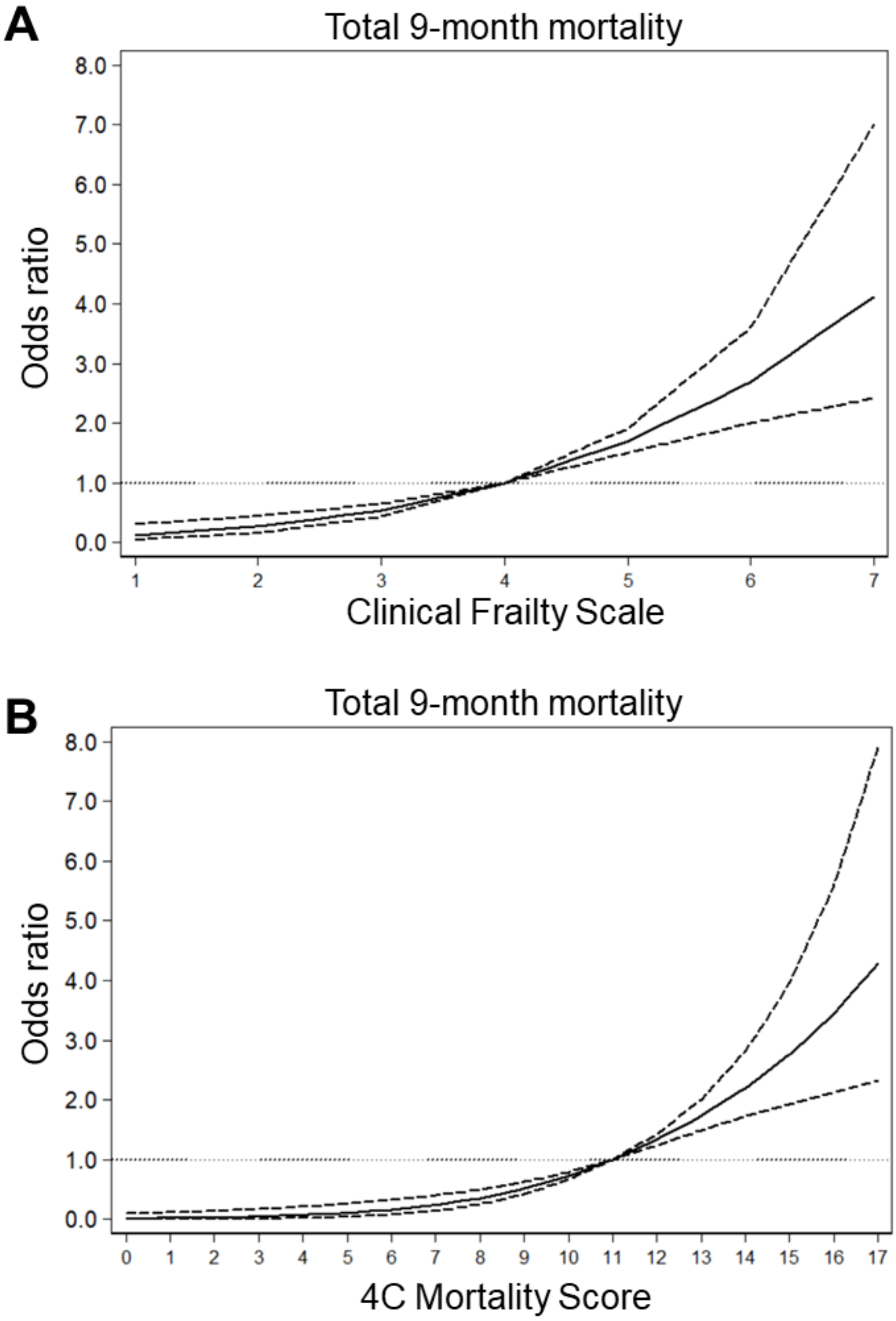

Figure S1. Smoothed restricted cubic spline plots of the odds ratio (OR) for overall mortality in patients with COVID-19 versus the baseline levels of **A.** Clinical Frailty Scale (CFS) and **B.** 4C mortality score. Depicted odds ratios indicate comparisons to the reference point (median value of CFS or 4C in our cohort). To enhance visual clarity, graphs were limited to the 95th percentile of corresponding scores. Three knots were fixed at the 10th, 50^{th,} and 90th percentile of CFS and 4C distribution. The upper- and lower-most dashed curves represent the 95% CI of the predicted ORs (middle solid line).

Figure S2

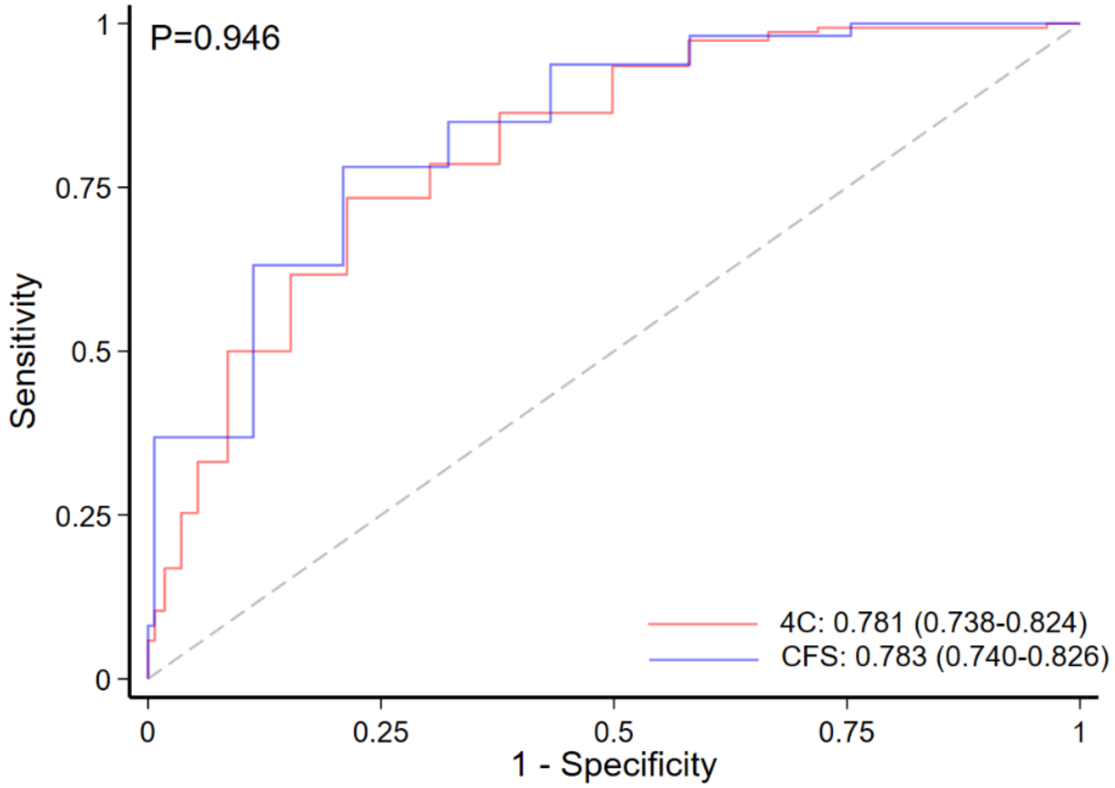

Figure S2: Receiver operating characteristic (ROC) curves and corresponding area(s) under the curve (AUC) for total 9-month mortality in patients hospitalized with COVID-19. The discriminatory performance of the 4C Mortality and the Clinical Frailty Scale score for the occurrence of death is graphically assessed by corresponding ROC curves and AUCs are compared to derive P-values using the chi-square distribution with one degree of freedom.
